# Non-genotoxic conditioning facilitates hematopoietic stem cell gene therapy for hemophilia A using bioengineered factor VIII

**DOI:** 10.1016/j.omtm.2021.04.016

**Published:** 2021-05-05

**Authors:** Athena L. Russell, Chengyu Prince, Taran S. Lundgren, Kristopher A. Knight, Gabriela Denning, Jordan S. Alexander, Jaquelyn T. Zoine, H. Trent Spencer, Shanmuganathan Chandrakasan, Christopher B. Doering

**Affiliations:** 1 Graduate Program in Genetics and Molecular Biology, Laney Graduate School, Emory University, Atlanta, GA 30322, USA; 2 Aflac Cancer and Blood Disorders Center, Department of Pediatrics, Emory University School of Medicine, Atlanta, GA 30322, USA; 3 Graduate Program in Molecular and Systems Pharmacology, Laney Graduate School, Emory University, Atlanta, GA 30322, USA; 4 Expression Therapeutics, LLC, Tucker, GA 30084, USA; 5 Graduate Program in Cancer Biology, Laney Graduate School, Emory University, Atlanta, GA 30322, USA

**Keywords:** non-genotoxic conditioning, antibody conditioning, immunotoxin, antibody-drug conjugate, hematopoietic stem cell transplantation, *ex vivo* lentiviral vector gene therapy, hemophilia A, factor VIII

## Abstract

Hematopoietic stem and progenitor cell (HSPC) lentiviral gene therapy is a promising strategy toward a lifelong cure for hemophilia A (HA). The primary risks associated with this approach center on the requirement for pre-transplantation conditioning necessary to make space for, and provide immune suppression against, stem cells and blood coagulation factor VIII, respectively. Traditional conditioning agents utilize genotoxic mechanisms of action, such as DNA alkylation, that increase risk of sterility, infection, and developing secondary malignancies. In the current study, we describe a non-genotoxic conditioning protocol using an immunotoxin targeting CD117 (c-kit) to achieve endogenous hematopoietic stem cell depletion and a cocktail of monoclonal antibodies to provide transient immune suppression against the transgene product in a murine HA gene therapy model. This strategy provides high-level engraftment of hematopoietic stem cells genetically modified *ex vivo* using recombinant lentiviral vector (LV) encoding a bioengineered high-expression factor VIII variant, termed ET3. Factor VIII procoagulant activity levels were durably elevated into the normal range and phenotypic correction achieved. Furthermore, no immunological rejection or development of anti-ET3 immunity was observed. These preclinical data support clinical translation of non-genotoxic antibody-based conditioning in HSPC LV gene therapy for HA.

## Graphical abstract

**Figure:**
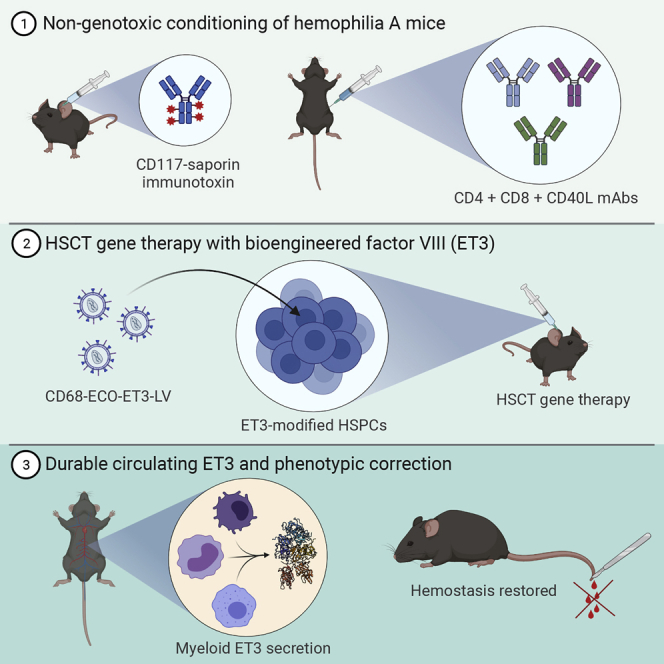

## Teaser

Doering and colleagues develop a non-genotoxic conditioning regimen that permits successful long-term engraftment of hematopoietic stem and progenitor cells genetically modified with a high-expression factor VIII variant in hemophilia A mice. Durable factor VIII expression and hemostatic correction are achieved through non-genotoxic hematopoietic stem cell transplantation gene therapy.

## Introduction

Hemophilia A (HA) is the most common severe congenital bleeding disorder caused by a deficiency in blood coagulation factor VIII (fVIII) due to mutations in the *F8* gene. The disease affects approximately 1:4,000 male births and is associated with an elevated risk of bleeding-related mortality. In severe cases (less than 1% fVIII activity), persons with HA suffer spontaneous bleeding into joints leading to progressive, debilitating arthropathy, as well as life-threatening bleeds into closed spaces such as intracranial or retroperitoneal cavities.^1^ Standard of care involves either prophylactic fVIII replacement or fVIII mimetic therapy. Although largely effective, these existing therapies are economically challenging, if not completely prohibitive, for the global majority. Furthermore, even with optimal therapy, bleeding and joint damage/pain still occur and unmet clinical need exists.^2^^,^^3^

HA has a long-standing history with experimental gene therapies.^4^^,^^5^ It is likely that sustained, steady-state production and circulation of fVIII would overcome most of the limitations of existing replacement, mimetic, or bypassing therapies. It has been established in animal models and clinical trials that modest increases in circulating fVIII activity can lead to clinically significant reduction in bleeding challenge assays and annualized bleed rate, respectively. Furthermore, due to the lack of cell-type-specific post-translational modifications (e.g., gamma carboxylation), virtually any cell type can biosynthesize and secrete functional fVIII into the circulation. Gene transfer strategies employing adeno-associated viral (AAV) vectors to target liver hepatocytes or lentiviral vectors (LVs) targeting hematopoietic stem and progenitor cells (HSPCs) *ex vivo* have advanced into clinical testing. Multiple AAV-fVIII gene therapies have already progressed into phase 3 clinical studies, although concerns remain regarding long-term safety and durability. In contrast, HSPC LV-fVIII gene therapies are just beginning phase 1/2 testing, and no clinical data have been released. Additionally, systemic *in vivo* LV delivery targeting hepatocytes for HA gene therapy is in preclinical development. This approach has potential advantages over *in vivo* AAV delivery (e.g., vector integration allows stable transgene expression, even during substantial organ growth that occurs in children, and the low prevalence of antibodies against LV in the general population). However, many questions remain to be answered to ensure the safety and feasibility of this approach. Although vesicular stomatitis virus surface glycoprotein (VSV-G), the most common LV pseudotype, allows for considerable tropism to the liver,^6^ VSV-G-LVs have extremely broad tropism,^7^ and since genotoxicity remains an inherent risk with integrating vectors, extensive biodistribution studies will be needed. Challenges associated with vector quantity and quality will also need to be overcome before LVs can be administered systemically in humans.^6^

*Ex vivo* LV modification of autologous HSPCs followed by transplantation (HSCT) is a promising approach for gene therapy of HA^8^^,^^9^ and other monogenic blood and immune cell diseases.^10^^,^^11^ Since HSPCs are responsible for producing a lifelong supply of blood cells, gene replacement strategies that target these long-term repopulating cells offer the potential for continuous production of fVIII from daughter cells and correction of disease phenotypes for the lifetime of the individual. Our laboratory has created a bioengineered high-expression fVIII variant, termed ET3, that has been optimized for recombinant LV delivery to HSPCs.^12^, ^13^, ^14^, ^15^, ^16^, ^17^ Expression of the codon-optimized ET3 transgene is driven by the CD68 promoter, enabling high-level monocyte-lineage restricted expression of ET3 upon HSPC differentiation.^18^ ET3 is secreted by gene-modified monocyte and macrophage populations into the bloodstream, and sustained plasma ET3 levels thereby confer lifelong hemostatic correction.

HSCT is a powerful and potentially curative therapy used for a broad range of malignant and non-malignant diseases,^19^ with its therapeutic potential further amplified when combined with gene therapy. However, the clinical impact of HSCT gene therapy is primarily limited by the cytotoxic and genotoxic effects of conventional conditioning agents, such as total body irradiation (TBI) and alkylating chemotherapeutics, which are DNA damaging.^20^ Significant acute and long-term toxicities and treatment-related mortality are associated with non-targeted, genotoxic conditioning regimens, including organ toxicity, infertility, and risk of secondary malignancy.^21^, ^22^, ^23^, ^24^ Myeloablative conditioning regimens cause cytopenias in the early post-transplant period before engraftment of leukocyte and platelet populations, leaving the patient vulnerable to infection and bleeding.^19^^,^^25^^,^^26^ Thrombocytopenia and attendant increased bleeding risk caused by genotoxic conditioning is particularly undesirable in the setting of HA. Therefore, the development of alternative, non-genotoxic conditioning agents and regimens is a high-priority translational objective for the fields of HSCT and HSPC-directed gene therapy.

## Results

### Generation of saporin-based anti-mouse CD117 immunotoxin (CD117-sap)

For this study, we generated an immunotoxin using a monoclonal antibody (mAb) specific for the stem cell factor (SCF) receptor CD117 (clone 2B8) present on mouse HSPCs as the targeting moiety, coupled to the type I ribosome-inactivating protein toxin saporin. Commercially available biotinylated CD117 mAb was linked to a streptavidin-saporin conjugate that was either purchased commercially (see Supplemental materials) or generated in-house (Figure S1A). Commercial saporin is isolated directly from seeds of the *Saponaria officinalis* plant, whereas our in-house product is a recombinant version.^27^ Our laboratory has optimized the production and purification of recombinant saporin, as well as the bioconjugation of saporin to streptavidin for immunotoxin generation. Figure S1B shows a representative Coomassie-stained polyacrylamide gel of biotinylated CD117 mAb, commercial saporin, in-house-generated recombinant saporin, and their associated CD117-sap products after conjugation.

### CD117-sap selectively depletes hematopoietic stem cells (HSCs) and spares non-hematopoietic tissues in HA mice

**Figure 1:**
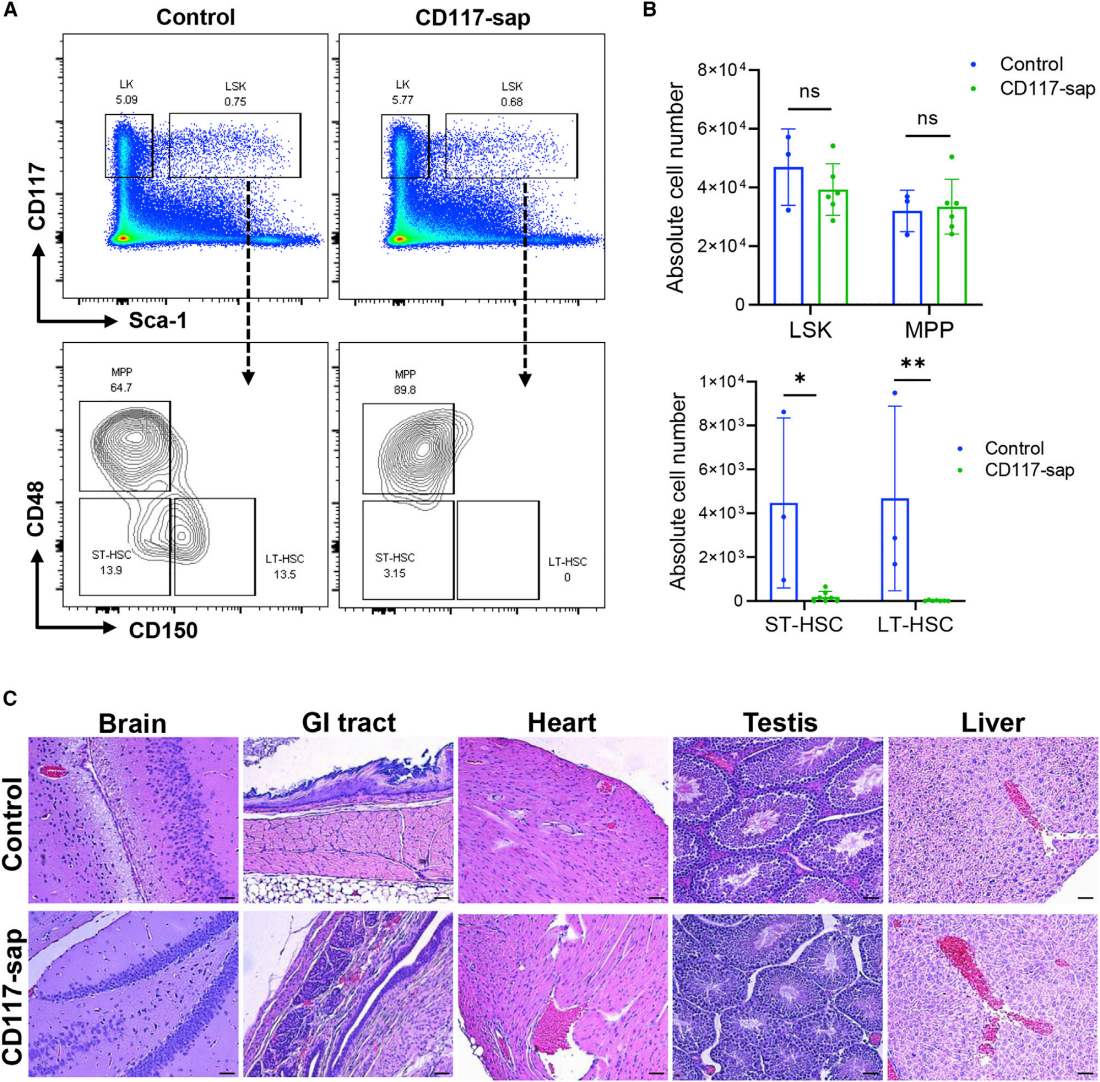
CD117-saporin immunotoxin selectively depletes HSCs and spares non-hematopoietic tissues in hemophilia A (HA) mice (A) Representative flow cytometry plots of bone marrow demonstrate selective and robust depletion of short-term (LSK CD48^−^ CD150^−^) and long-term (LSK CD48^−^ CD150^+^) HSC compartments 5 days post-conditioning with CD117-sap. (B) Quantification of HSPC depletion after treatment with CD117-sap immunotoxin (n = 6) compared with no immunotoxin (n = 3) illustrates selective HSC depletion. Data represent mean ± sample SD. Statistical comparison performed using Mann-Whitney U test. ∗p < 0.05, ∗∗p < 0.01, ns = not significant. (C) H&E staining of non-hematopoietic tissues known to express CD117 revealed no evidence of toxicity or histopathological lesions. Scale bars: 100 μm.

We first evaluated the ability of CD117-sap immunotoxin to deplete HSCs in HA mice, thereby creating space in bone marrow niches for engraftment of ET3 gene-modified donor HSPCs. HA mice were conditioned with 0.5 mg/kg CD117-sap, and bone marrow was evaluated for depletion of HSPC subsets by flow cytometry. Although CD117-expressing hematopoietic progenitor cell (HPC) populations, such as Lineage (Lin)^−^ Sca-1^+^ CD117^+^ (LSK) and Lin^−^ Sca-1^+^ CD117^+^ CD48^+^ CD150^−^ (MPP) compartments, were minimally affected 5 days after CD117-sap treatment, we observed robust and specific depletion of the short-term (ST-HSC) and long-term repopulating HSC (LT-HSC) populations (Lin^−^ Sca-1^+^ CD117^+^ CD48^−^ CD150^−^ and Lin^−^ Sca-1^+^ CD117^+^ CD48^−^ CD150^+^, respectively) compared with control mice that received no immunotoxin (Figures 1A and 1B). C57BL/6 mice were conditioned with 0.5 mg/kg CD117-sap at various time points and transplanted with 5 × 10^6^ congenic whole bone marrow cells to determine optimal timing of immunotoxin conditioning relative to transplantation. CD117-sap conditioning 5 days before transplantation led to 90.6% ± 5.9% donor myeloid chimerism at 4 weeks post-transplantation that increased to 94.5% ± 3.3% by 16 weeks (Figure S2). Based on these observations, a 5-day conditioning period was selected for all future studies.

Although expression of CD117 is found predominantly in the HSPC compartment, certain non-hematopoietic cells and tissues express some level of CD117, such as Cajal cells of the gastrointestinal tract, germ cells, and some neurons.^28^ Histological analysis of various CD117^+^ tissues in conditioned mice and untreated controls was performed 5 days after conditioning to investigate whether CD117-sap immunotoxin elicited toxicity in healthy CD117^+^ tissues outside the hematopoietic compartment. No evidence of gross toxicity or pathological lesions in any tissues was observed by necropsy or histology following CD117-sap treatment (Figure 1C). Collectively, these data illustrate that CD117-sap immunotoxin conditioning potently depletes ST- and LT-HSC populations in HA mice without causing overt toxicity in non-target tissues.

### CD117-sap plus anti-thymocyte globulin enables engraftment of ET3-modified LT-HSCs in a subset of mice

We next evaluated whether CD117-sap immunotoxin could effectively condition the bone marrow of HA mice to engraft congenic donor HSPCs modified using an LV harboring our expression codon-optimized (ECO) ET3 transgene driven by the CD68 promoter (CD68-ECO-ET3-LV). We have previously shown that host immune suppression using T cell costimulatory blocking agents or anti-thymocyte serum was necessary to achieve high donor chimerism of gene-modified cells and sustained fVIII activity in the absence of inhibitor development in LV-HSCT studies using various non-myeloablative conditioning regimens and a B-domain deleted porcine fVIII transgene.^1^ These observations informed our hypothesis that transient immune suppression would likely be necessary to enable long-term engraftment of ET3 gene-modified HSPCs and durable fVIII expression in the context of non-genotoxic immunotoxin conditioning.

**Figure 2:**
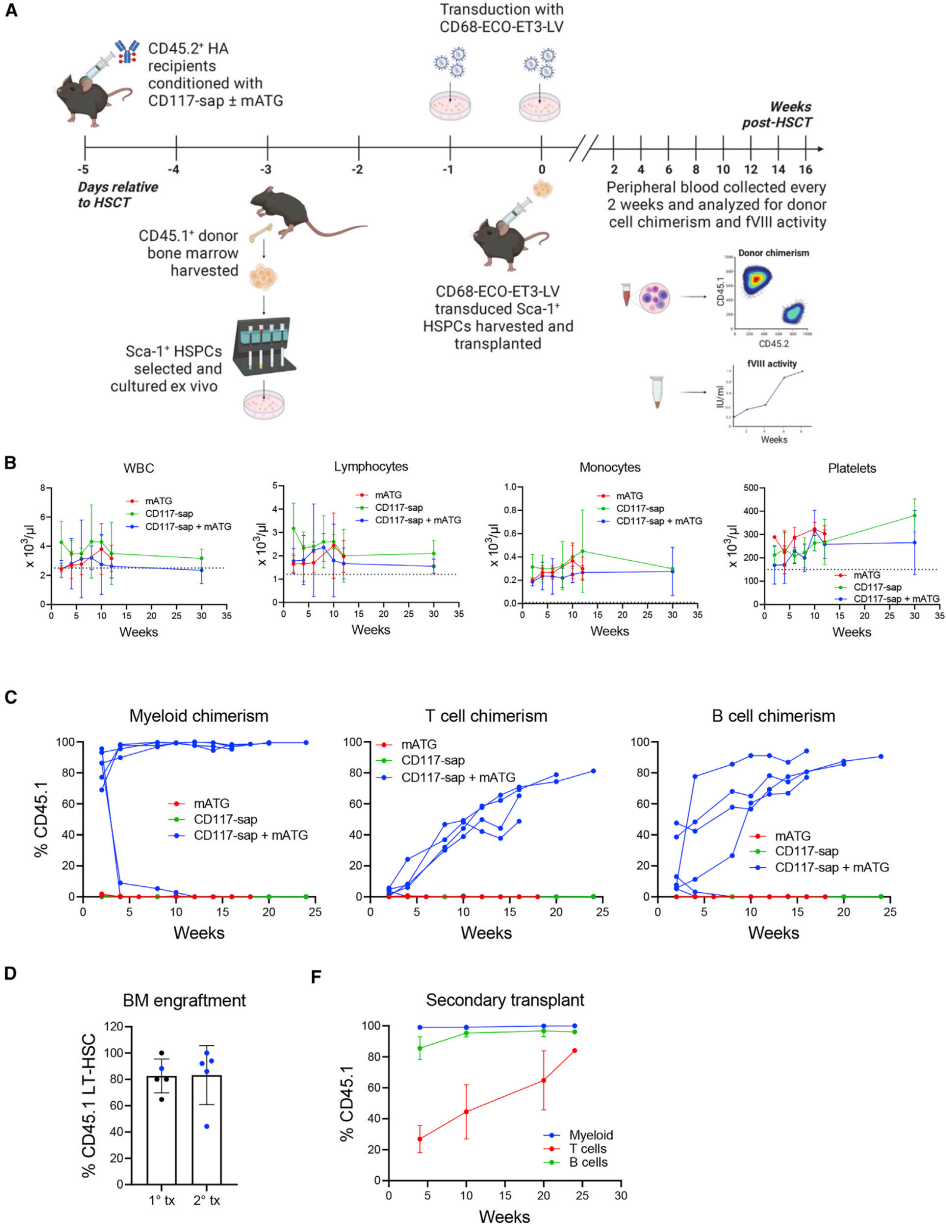
CD117-sap + mATG conditioning enables engraftment of CD68-ECO-ET3-LV-transduced LT-HSCs and multilineage chimerism in a majority of recipients (A) Schematic of experimental design illustrates timeline of CD117-sap + mATG conditioning followed by transplantation of ET3 gene-modified Sca-1^+^ cells in HA mice. (B) CD117-sap + mATG conditioning (n = 7) did not produce prolonged leukopenia or lymphopenia in the early post-transplantation period. No detrimental effects to monocyte populations were observed. Platelet counts were preserved in the early post-transplantation period. Dotted line represents lower limit of reference range for each population. Data represent mean ± sample SD. See also Figures S3B and S4. (C) High-level early myeloid chimerism was observed in 6 out of 7 mice conditioned with CD117-sap + mATG 2 weeks post-transplantation. Multilineage hematopoietic chimerism of donor-derived cells was sustained for up to 30 weeks in 4 out of 7 HA recipients conditioned with CD117-sap + mATG. Conditioning with CD117-sap or mATG alone did not permit engraftment. See also Figure S5. (D) Donor-derived LT-HSCs engrafted in bone marrow of mice conditioned with CD117-sap + mATG (n = 5) for primary transplant (1° tx) and secondary transplant (2° tx) recipients (n = 5). Blue dots represent primary recipient that acted as donor for respective secondary recipients. Data represent mean ± sample SD. See also Figure S6A. (E) Secondary HA recipients (n = 5) conditioned with 9 Gy total body irradiation were serially transplanted with whole bone marrow from a primary HA recipient conditioned with CD117-sap + mATG and followed for 24 weeks. Results demonstrate that immunotoxin conditioning enabled engraftment of donor-derived long-term repopulating HSCs in primary recipients. Data represent mean ± sample SD. See also Figure S7A.

CD45.2^+^ HA mice were conditioned with 0.5 mg/kg CD117-sap with or without 30 mg/kg mouse anti-thymocyte globulin (mATG), or mATG alone, and then transplanted 5 days later with 1 × 10^6^ CD68-ECO-ET3-LV-transduced CD45.1^+^ Sca-1^+^ HSPCs. Mice conditioned with TBI were included as positive controls to ensure viability and engraftment potential of the HSPCs following *ex vivo* culture and transduction. Depletion of ST-HSC and LT-HSC compartments in mice conditioned with CD117-sap + mATG were analyzed, and findings were consistent with patterns observed in mice conditioned with CD117-sap alone (Figure S3A). Peripheral blood and plasma were collected every 2 weeks, and complete blood counts (CBCs), multilineage donor cell chimerism, and ET3 activity were measured (Figure 2A). Conditioning with CD117-sap + mATG permitted normal white blood cell (WBC) and lymphocyte counts within 2–4 weeks after transplantation and did not produce prolonged leukopenia. Platelet counts were also preserved in CD117-sap + mATG conditioned mice, and monocyte counts were not negatively impacted by the conditioning regimen (Figures 2B and S3B). In a separate experiment, leukocyte and lymphocyte subpopulations following mATG administration were tracked for 21 days post-conditioning to examine kinetics of depletion and recovery (Figure S4). Two weeks after transplantation, 1 of 7 mice (across two independent experimental cohorts) conditioned with CD117-sap + mATG failed to engraft ET3 gene-modified HSPCs (Figure S5A, orange diamond), while the remaining six mice achieved early myeloid chimerism of 72.5% ± 33.3% (mean ± sample standard deviation [SD]). Mice receiving CD117-sap alone or mATG alone universally failed to engraft gene-modified cells (Figure 2C). By 4 weeks post-transplantation, myeloid chimerism levels decreased from 95.6% to 0.22% and 86.4% to 8.94% in two additional mice conditioned with CD117-sap + mATG (Figure S5A, red square and brown square). The remaining four mice in this group maintained high levels of donor myeloid chimerism up to 24 weeks post-transplantation. T and B cell chimerism continued to rise in these mice for the duration of the study (Figures 2C and S5). Upon sacrifice at 30 weeks post-transplantation, engraftment of donor-derived LT-HSCs in chimeric HA mice conditioned with CD117-sap + mATG was 80.2% ± 16.1% (Figures 2D and S6A). For comparison of the CD117-sap + mATG regimen with another non-myeloablative regimen, in a separate set of experiments HA mice were conditioned with three doses of 15 mg/kg busulfan and two doses of 20 mg/kg mATG before transplantation with 1 × 10^6^ CD68-ECO-ET3-LV-transduced CD45.1^+^ Sca-1^+^ HSPCs. TBI-conditioned mice were included as controls for comparison of donor chimerism and fVIII activity. Eight weeks post-transplantation, mice conditioned using CD117-sap + mATG with sustained engraftment achieved higher mean donor leukocyte chimerism than mice conditioned with the busulfan + mATG regimen (73.2% ± 13.9% versus 60.1% ± 11.0%, respectively) (Figure S6B).

Bone marrow from one primary recipient conditioned with CD117-sap + mATG (95.2% myeloid chimerism, 88.2% LT-HSC chimerism) and one conditioned with TBI (98.8% myeloid chimerism, 97.4% LT-HSC chimerism) was harvested and transplanted into lethally irradiated secondary HA recipients (n = 5 secondary recipients). Early CD45.1^+^ myeloid chimerism in secondary recipients reached 99.0% ± 0.7% 4 weeks after serial transplantation and remained at this level for 24 weeks. B cell CD45.1^+^ chimerism reached 95.4% ± 2.5% by 8 weeks and was sustained, while T cell CD45.1^+^ chimerism continued to increase throughout the study (Figure 2E). Results were similar in secondary recipients of TBI donor marrow (Figure S7A). CD45.1^+^ LT-HSC engraftment was 83.3% ± 22.4% in secondary recipients receiving CD117-sap + mATG-conditioned bone marrow and 99.9% ± 0.06% for secondary recipients receiving TBI-conditioned bone marrow (Figures 2D and S6A), which were consistent with levels measured in the primary recipients.

Taken together, these data demonstrate that CD117-sap + mATG enabled high-level engraftment of CD68-ECO-ET3-LV modified donor LT-HSCs in the absence of prolonged lymphopenia in a majority of HSCT recipients. However, 43% (3 out of 7) of mice conditioned with CD117-sap + mATG did not maintain long-term donor engraftment. Despite this variability, immune suppression with mATG was required for successful engraftment of ET3 gene-modified cells in HA mice.

### CD117-sap + mATG conditioning promotes therapeutic and durable ET3 expression

**Figure 3:**
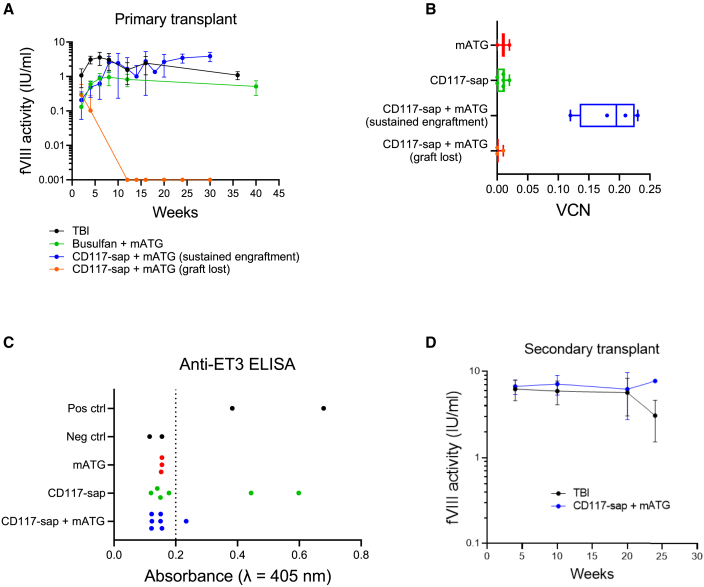
HA mice engrafted with gene-modified HSCs following CD117-sap + mATG conditioning exhibited therapeutic levels of plasma ET3 activity while some non-engrafted mice developed ET3 inhibitors (A) ET3 activity was determined by chromogenic assay in HA mice that maintained long-term engraftment of ET3 gene-modified HSCs (TBI, n = 9; Busulfan + mATG, n = 5; CD117-sap + mATG, n = 4; CD117-sap + mATG [graft lost], n = 3). Data represent mean ± sample SD. (B) Vector copy number was measured by real-time PCR of proviral DNA in peripheral blood of primary recipients 10 weeks post-transplantation (mATG, n = 3; CD117-sap, n = 6; CD117-sap + mATG, n = 4; CD117-sap + mATG [graft lost], n = 3). See also Figure S7B. (C) Anti-ET3 antibodies were detected by ELISA in three mice 14 weeks post-transplantation. Dotted line represents threshold for positive signal. Inhibitors were detected in two mice that failed to engraft gene-modified HSCs following conditioning with CD117-sap alone. One mouse conditioned with CD117-sap + mATG did not have sustained engraftment and developed ET3 inhibitors. Bethesda titers shown in Figure S7C. (D) Sustained high levels of ET3 activity measured in lethally irradiated HA secondary recipients following serial transplantation indicate engraftment of gene-modified, LT-HSCs after TBI (n = 5 secondary recipients) or CD117-sap + mATG (n = 5 secondary recipients) conditioning in primary recipients. Data represent mean ± sample SD.

Next, we examined whether high-level engraftment of ET3 gene-modified HSPCs could produce sustained therapeutic levels of ET3 hemostatic activity in HA recipients after CD117-sap + mATG conditioning. We then compared these results with those obtained using the non-myeloablative busulfan + mATG regimen or TBI. Plasma was collected and tested by chromogenic assay. ET3 activity in mice conditioned with CD117-sap + mATG that sustained long-term donor chimerism measured 0.21 ± 0.15 IU/mL (21% the normal human level) and 0.13 ± 0.18 IU/mL (13% of normal) in busulfan + mATG mice as early as 2 weeks post-HSCT. By 6 weeks, curative levels of 0.88 ± 0.35 IU/mL (88% of normal) were achieved in the busulfan + mATG group, and 0.62 ± 0.40 IU/mL (62% of normal) using CD117-sap + mATG conditioning, with ET3 activity continuing to increase for the duration of the study in the latter group. Mice conditioned with CD117-sap + mATG that did not maintain donor chimerism also lost fVIII activity (Figure 3A).

Vector copy number (VCN) of CD68-ECO-ET3-LV was measured in DNA isolated from peripheral blood of mATG-, CD117-sap-, or CD117-sap + mATG-treated mice 10 weeks post-transplantation. Peripheral blood vector copy number in mice conditioned with CD117-sap + mATG that maintained the graft was 0.19 ± 0.05 copies per genome (Figures 3B and S7B). ELISA assays were performed to detect the presence of anti-ET3 immunoglobulin G (IgG) antibodies to determine whether mice conditioned with mATG, CD117-sap alone, or CD117-sap + mATG that failed to durably engraft gene-modified HSPCs also mounted a humoral response against the ET3 transgenic protein. Although none of the six mice conditioned with CD117-sap alone without mATG immune suppression successfully engrafted ET3 gene-modified HSPCs at any point during the study, only two developed measurable levels of anti-ET3 antibodies (Figure 3C). Similarly, of the three mice conditioned with CD117-sap + mATG that failed to maintain engraftment during the study, only one developed antibodies against ET3. Bethesda assays were performed to quantify titers in mice that developed ET3-neutralizing inhibitors (Figure S7C). The immunological mechanism responsible for rejection of ET3 gene-modified cells in mice that did not produce ET3 inhibitors was not identified.

ET3 activity in secondary HA recipients was measured by chromogenic assay for 24 weeks after serial transplantation. Levels of ET3 activity reached 6.7 ± 1.3 IU/mL (660% of normal) 4 weeks after secondary HSCT with CD117-sap + mATG donor marrow and were sustained for the duration of the study (Figure 3D). These findings illustrate that CD68-ECO-ET3-LV HSPC-directed gene therapy using non-genotoxic CD117-sap + mATG conditioning enables durable therapeutic levels of ET3 activity in HA mice following engraftment of gene-modified cells. However, in approximately one-third of mice that did not maintain long-term engraftment under CD117-sap or CD117-sap + mATG conditioning, an immune response to ET3 was observed.

### mATG exhibits a broad-ranging binding profile that includes mature and primitive hematopoietic cells

In an effort to identify potential causes for why certain mice conditioned with CD117-sap + mATG failed to engraft or maintain engraftment of ET3 gene-modified cells, we turned our attention to mATG. The mATG used in our study was produced by immunizing rabbits with mouse thymocytes and collecting the IgG fraction. Human ATG products are known to contain a multitude of antibodies with varying specificities, with new target antigens being continually identified that can be found on a variety of cell types and involved in diverse cellular pathways.^29^^,^^30^ With concerns over whether residual mATG in circulation at the time of HSCT could potentially bind donor HSCs and interfere with engraftment, we aimed to characterize the binding profile of the mATG reagent.

**Figure 4:**
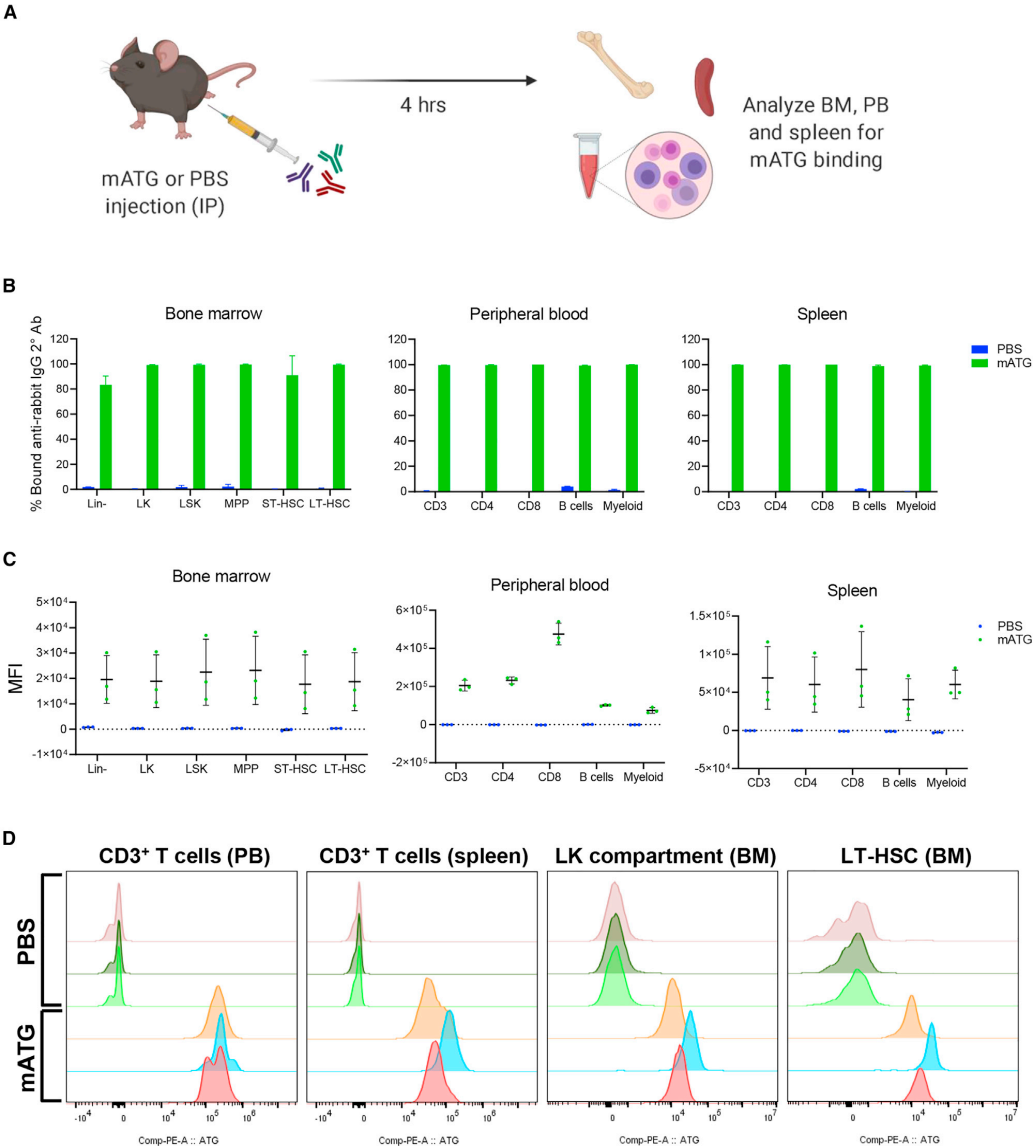
Rabbit anti-mouse mATG binds mature and primitive hematopoietic cells in peripheral blood, spleen, and bone marrow (A) Schematic illustrates experimental design of *in vivo* mATG binding study. (B) mATG binding to various cell populations in each tissue was detected by flow cytometry using an anti-rabbit IgG secondary antibody. Data represent mean ± sample SD (n = 3 in each group). See also Figure S9A. (C) Median fluorescence intensity (MFI) of mATG staining reveals greatest intensity of binding on CD8^+^ peripheral blood T cell subset. MFIs measured in bone marrow subsets were 10- to 20-fold lower than in peripheral blood subsets. See also Figure S9B. (D) Flow cytometry histograms exemplify mATG binding on spleen and peripheral blood (PB) T cells, Lin^−^ CD117^+^ (LK) HSPCs, and LT-HSCs in bone marrow (BM). See also Figure S8.

HA mice were injected intraperitoneally (i.p.) with 30 mg/kg mATG and sacrificed after 4 h (Figure 4A). Blood, spleen, and bone marrow cells were harvested and stained with an anti-rabbit IgG secondary antibody to detect cells bound by mATG and then stained for LSK-SLAM markers in bone marrow or mature lineage markers in spleen and blood. Flow cytometric analysis revealed substantial mATG binding to every cell population examined in all three tissues (Figure 4B). As expected, mATG antibodies were extensively bound to T cell subsets, yet mATG was also detected on B cells and Gr-1^+^ myeloid cells. Furthermore, considerable binding of mATG antibodies was detected on all lineage-negative bone marrow subsets evaluated, including ST- and LT-HSC populations. Median fluorescence intensity (MFI) of mATG staining (as measured by secondary antibody signal) was greatest in the peripheral blood CD8^+^ T cell compartment, while MFIs of the various bone marrow HSPC compartments were at least an order of magnitude lower than those measured from peripheral blood T cells (Figure 4C). Nevertheless, clear signals indicating mATG binding to HPC and HSC populations were observed (Figures 4D and S8). Similar results were obtained from binding studies using peripheral blood, bone marrow, and spleen cells from HA mice incubated with mATG *in vitro* and analyzed by flow cytometry (Figures S9A and S9B). Additionally, in transplantation studies, when mATG was administered the day before and the day of transplant, engraftment of transplanted HSPCs was abrogated (Figure S9C, red line). These results suggest off-target effects of mATG, potentially through direct interaction with the transplanted gene-modified HSPCs, likely confounded the previous studies and contributed to the variability in outcomes observed.

### CD117-sap plus T cell-targeted immune suppression invariably enables engraftment of ET3-modified LT-HSCs

**Figure 5:**
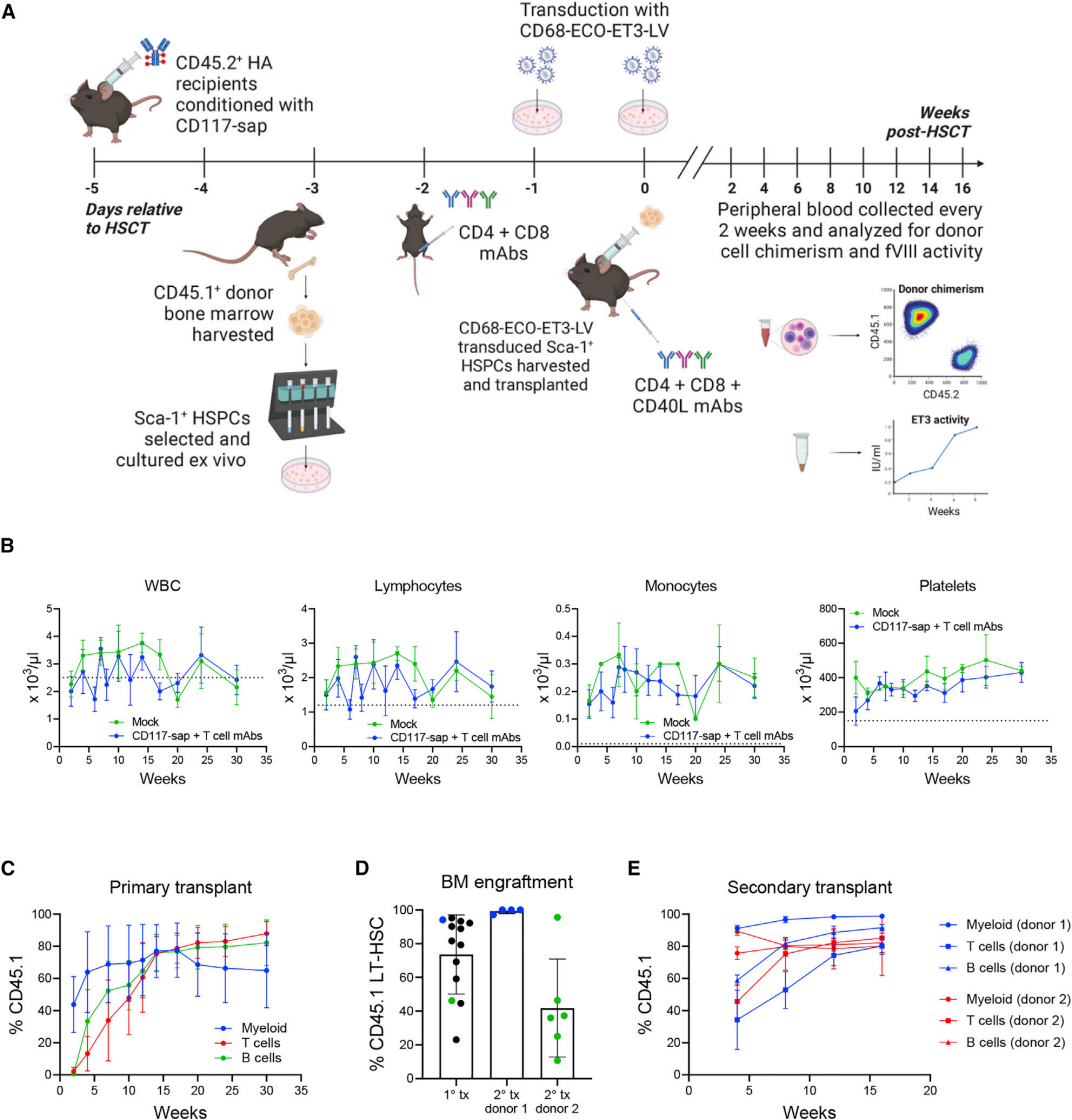
CD117-sap + T cell mAb conditioning enables engraftment of CD68-ECO-ET3-LV gene-modified LT-HSCs and multilineage hematopoietic chimerism in all transplant recipients (A) Schematic of experimental design illustrates timeline of CD117-sap + T cell mAb conditioning followed by transplantation of ET3 gene-modified Sca-1^+^ cells in HA mice. (B) CD117-sap + T cell mAb (ET3-transduced cell transplant, n = 13; mock-transduced cell transplant, n = 3) conditioning did not produce prolonged leukopenia or lymphopenia in the early post-transplant period. No detrimental effects to monocyte populations were observed. Platelet counts were preserved in the early post-transplantation period in mice conditioned with CD117-sap + T cell mAbs. Dotted line represents lower limit of reference range for each population. Data represent mean ± sample SD. See also Figures S10A and S4. (C) Multilineage hematopoietic chimerism of donor-derived cells was sustained for up to 30 weeks in 100% of HA recipients conditioned with CD117-sap + T cell mAbs (n = 13). Data represent mean ± sample SD. See also Figure S10B. (D) Donor-derived LT-HSCs engrafted in bone marrow of mice conditioned with CD117-sap + T cell mAbs (n = 13) for the primary transplant (1° tx) and secondary transplant (2° tx) recipients from two donors (n = 6 per donor). Data represent mean ± sample SD. Blue and green dots represent donors for respective secondary transplant recipients. See also Figure S11A. (E) Secondary HA recipients (n = 6 in each group) conditioned with 9 Gy total body irradiation were serially transplanted with whole bone marrow from two primary HA recipients conditioned with CD117-sap + T cell mAbs and followed for 16 weeks. Results demonstrate that immunotoxin conditioning enabled engraftment of donor-derived long-term repopulating HSCs in primary recipients. Data represent mean ± sample SD.

Immune suppression is a requirement for engraftment of CD68-ECO-ET3-LV gene-modified cells. Although there are important aspects of mATG immune suppression that enabled engraftment and ET3 expression in a majority of recipients, there appeared to be additional aspects that could also be interfering. We aimed to develop a more targeted approach to immune suppression in our gene therapy model in an attempt to replicate the important aspects of mATG mechanism of action on immune suppression while excluding potential confounding effects relating to the polyvalent and non-thymocyte-specific nature of mouse-specific ATG. Since the immune response to fVIII is T cell dependent,^31^^,^^32^ we designed a naked monoclonal antibody regimen specifically targeting T cells in order to control host T cell responses directed at ET3-modified cells. HA mice were conditioned with CD117-sap followed by antibody cocktails directed at CD4, CD8, and CD40L antigens (collectively referred to as T cell mAbs) and then transplanted with 2.5 × 10^6^ CD68-ECO-ET3-LV-transduced CD45.1^+^ Sca-1^+^ HSPCs (Figure 5A). CD4 (clone GK1.5) and CD8 (clone YTS169.4) mAbs produce *in vivo* depletion of respective T cell subsets, whereas CD40L mAb (clone MR-1) blocks CD40/CD40L signaling. CD40L is primarily expressed on activated CD4^+^ T cells, and the interaction of CD40L with CD40 expressed by antigen-activated B cells is critical for humoral immune responses against T cell-dependent antigens.^33^

This regimen permitted normal total lymphocyte counts by 2 weeks post-transplantation and normal monocyte and platelet counts throughout the study (Figures 5B and S10A). In a separate experiment, leukocyte and lymphocyte subpopulations following T cell mAb administration were tracked for 21 days post-conditioning to examine kinetics of depletion and recovery (Figure S4). Of 13 mice across two independent experimental cohorts, mice conditioned with CD117-sap + T cell mAbs uniformly maintained multilineage donor chimerism throughout the study up to 30 weeks post-transplantation, with no incidence of immune rejection (Figure 5C). Mean myeloid chimerism at 30 weeks was 64.9% ± 23.2% (range: 37.5%–99.9%), T cell chimerism was 88.0% ± 7.5% (range: 77.1%–98.0%), and B cell chimerism was 82.2% ± 14.2% (range: 63.6%–96.2%). A subgroup of mice that received CD117-sap + T cell mAb conditioning and mock-transduced Sca-1^+^ HSPCs achieved only slightly higher levels of donor chimerism in each cellular compartment (Figure S10B). Upon sacrifice, engraftment of donor-derived CD68-ECO-ET3-LV-transduced LT-HSCs in chimeric HA mice conditioned with CD117-sap + T cell mAbs was 73.6% ± 23.5% (range: 23.0%–95.2%) (Figures 5D and S11A).

Bone marrow from two primary recipients that received CD117-sap + T cell mAb conditioning and ET3-modified HSPCs (myeloid chimerism 97.9% and 76.9% and LT-HSC chimerism 94.1% and 46.2% when sacrificed at 16 weeks post-transplantation) was harvested and transplanted into lethally irradiated secondary HA recipients (n = 6 secondary recipients per donor). CD45.1^+^ myeloid chimerism in secondary recipients reached 98.8% ± 0.66% from donor 1 and 80.1% ± 18.3% from donor 2 after 16 weeks (Figure 5E, blue and red circles), consistent with levels measured in respective primary recipients at the same time point. Secondary recipients were sacrificed after 30 weeks, and CD45.1^+^ LT-HSC chimerism measured 99.3% ± 1.5% in recipients of donor 1 and 41.8% ± 29.1% in recipients of donor 2 (Figures 5D and S11A). These values were also consistent with LT-HSC chimerism measured in respective primary recipients.

Taken together, these data demonstrate that, although both regimens facilitate high multilineage engraftment in durably engrafted mice, CD117-sap + T cell mAb conditioning, in contrast to the CD117-sap + mATG regimen, enabled reliable engraftment of CD68-ECO-ET3-LV modified donor LT-HSCs in the absence of prolonged lymphopenia and with no incidence of immunological rejection in 100% of gene therapy recipients.

### Sustained therapeutic levels of ET3 activity uniformly achieved using CD117-sap + T cell mAb conditioning

**Figure 6:**
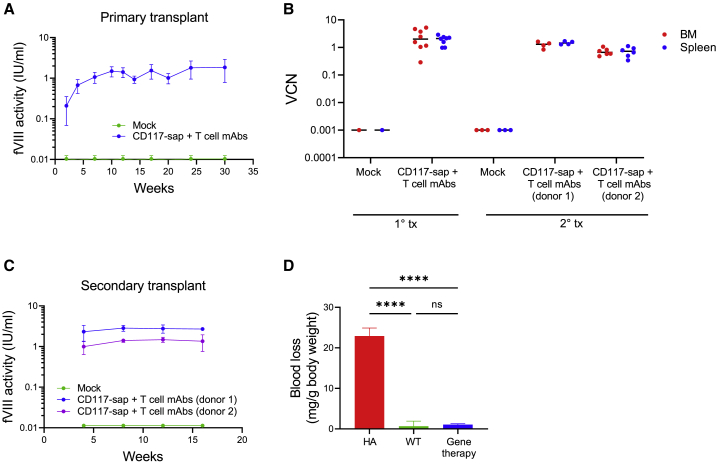
HA mice conditioned with CD117-sap + T cell mAbs and engrafted with CD68-ECO-ET3-LV gene-modified HSCs exhibited therapeutic levels of plasma ET3 activity and phenotypic correction (A) ET3 activity was determined by chromogenic assay of plasma from HA mice engrafted with ET3 gene-modified HSCs following CD117-sap + T cell mAb conditioning (n = 13) and mice that received CD117-sap + T cell mAb conditioning and mock-transduced HSCT (n = 3). Data represent mean ± sample SD. See also Figure S11B. (B) Vector copy number in primary (n = 8) and secondary (n = 4–6) recipients was measured by real-time PCR of proviral DNA from bone marrow and spleen. See also Figure S11C. (C) Sustained high levels of ET3 activity measured in lethally irradiated HA secondary recipients following serial transplantation indicate engraftment of ET3 gene-modified LT-HSCs in primary recipients (Mock, n = 3; CD117-sap + T cell mAbs, n = 6 in each group). Data represent mean ± sample SD. See also Figure S11B. (D) Correction of HA bleeding phenotype was observed using tail snip bleeding assay in gene therapy mice conditioned with CD117-sap + T cell mAbs (n = 3 in each group). Data represent mean ± sample SD. Statistical comparison performed using one-way ANOVA with Tukey’s multiple comparisons test. ∗∗∗∗p < 0.0001, ns = not significant.

Plasma samples were tested by chromogenic assay to ensure ET3 gene-modified HSCs engrafted under CD117-sap + T cell mAb conditioning could enable long-term therapeutic expression of circulating plasma ET3. Four weeks post-transplantation using the new targeted regimen, curative ET3 levels of 0.49 ± 0.31 IU/mL were achieved, steadily increasing to 1.76 ± 1.0 IU/mL by 30 weeks (Figures 6A and S11B). At the time of sacrifice, copies of CD68-ECO-ET3-LV proviral DNA were measured from bone marrow and spleen. In mice receiving the CD117-sap + T cell mAb regimen, vector copy number measured 2.5 ± 1.8 copies per genome in bone marrow and 1.9 ± 0.68 in spleen (Figures 6B and S11C).

ET3 activity in secondary HA recipients was measured by chromogenic assay for 16 weeks after serial transplantation. At the time of secondary transplantation, ET3 activity measured 2.60 IU/mL in donor 1 and 1.04 IU/mL in donor 2. Measured ET3 activity in secondary recipients was consistent with that measured in the donors; ET3 activity in secondary recipients of donor 1 bone marrow reached 2.70 ± 0.24 IU/mL, while activity in secondary recipients of donor 2 marrow reached 1.35 ± 0.59 IU/mL (Figures 6C and S11B). Vector copy number of secondary recipients was quantified from bone marrow and spleen at the time of sacrifice 30 weeks post-HSCT. Copies of CD68-ECO-ET3-LV proviral DNA in bone marrow measured 1.3 ± 0.33 (donor 1) and 0.71 ± 0.21 (donor 2), and spleen measured 1.47 ± 0.20 (donor 1) and 0.72 ± 0.29 (donor 2) (Figures 6B and S11C).

We performed a tail snip bleeding assay to assess correction of the characteristic bleeding phenotype of HA mice to demonstrate the functional efficacy of circulating plasma ET3 activity achieved from HSCT gene therapy under CD117-sap + T cell mAb conditioning. After 40 min of bleeding from a 4 mm excision from the tip of the tail, untreated HA control mice lost 22.9 ± 3.9 mg of blood per gram body weight. By contrast, wild-type fVIII-competent controls and mice treated with ET3 gene therapy bled significantly less, losing only 0.67 ± 2.2 and 1.1 ± 0.37 mg blood per gram body weight, respectively (Figure 6D). Together these data demonstrate that CD68-ECO-ET3-LV HSPC-directed gene therapy using non-genotoxic CD117-sap + T cell mAb conditioning enables long-term expression and functional activity of ET3, restoring hemostasis and correcting the disease phenotype in HA mice.

### Normal tissue morphology and fertility after engraftment of gene-modified HSCs and ET3 expression

**Figure 7:**
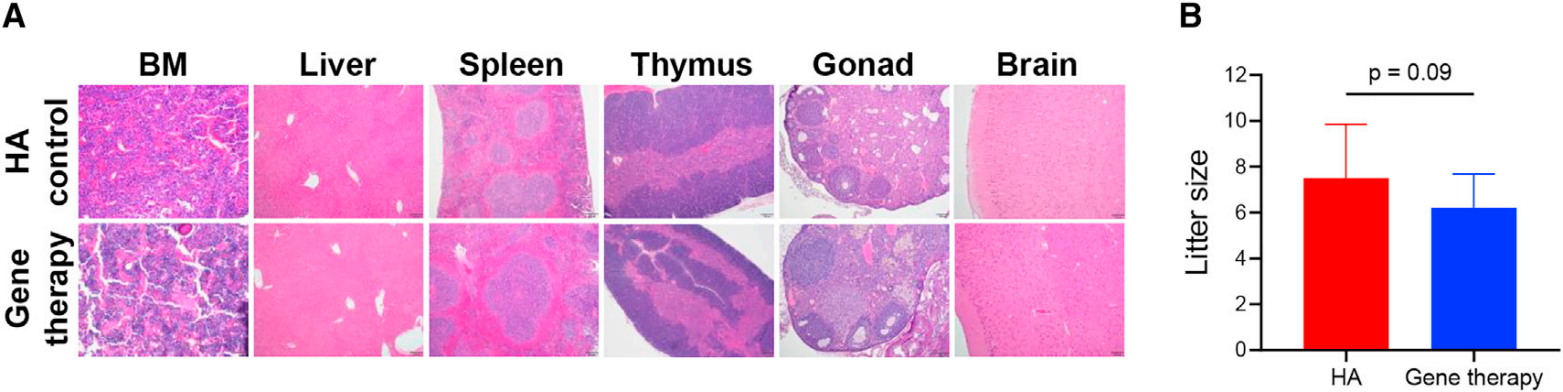
HA mice engrafted with ET3-modified cells after CD117-sap + T cell mAb conditioning exhibit healthy tissue morphology and normal litter sizes (A) H&E staining of various tissues revealed no evidence of toxicity or inflammatory processes following long-term engraftment of ET3 gene-modified cells. Scale bars: 50 μm (BM), 100 μm (all other tissues). (B) Average litter size was not significantly different between control HA breeder males (n = 12 litters) and HA males engrafted with ET3 gene-modified HSCs following CD117-sap + T cell mAb conditioning (n = 19 litters), indicating normal reproductive capacity following ET3 gene therapy with non-genotoxic conditioning. Statistical comparison performed using Student’s t test. Data represent mean ± sample SD.

Histological analysis of H&E-stained sections of various organs 34 weeks post-transplantation was performed to ensure the absence of organ toxicity following long-term engraftment of ET3-modified cells and CD68 promoter-driven ET3 expression from tissue-based macrophages. No evidence of toxicity, pathological lesions, or inflammatory processes was observed in liver, spleen, gonad, bone marrow, or brain due to ET3 gene therapy under CD117-sap + T cell mAb conditioning (Figure 7A). Six weeks after CD117-sap + T cell mAb conditioning and engraftment of ET3-modified HSCs, chimeric HA males were mated to untreated HA females to assess fertility and reproductive function. Chimeric males mated successfully and produced indistinguishable litter sizes compared to control HA breeder males (Figure 7B). Mating of chimeric HA males was followed up to 20 weeks post-engraftment, and no reproductive dysfunction was observed during this observation period.

## Discussion

Autologous HSC-directed gene therapy represents a promising strategy for providing a lifelong cure for HA. However, risks associated with DNA-damaging, genotoxic conditioning agents traditionally required for successful HSCT remain a concern. The preclinical findings described herein suggest that these risks can be mitigated. We demonstrate that endogenous HSCs can be specifically depleted in HA mice using a non-genotoxic immunotoxin targeting CD117 and, coupled with a non-genotoxic antibody cocktail targeting host T cells for immune suppression, HSCs modified to contain a functional copy of the fVIII variant ET3 can be successfully and durably engrafted with no signs of immunological rejection. Upon monocyte differentiation and tissue-based macrophage and dendritic cell maturation, sustained therapeutic levels of ET3 are secreted into plasma, restoring hemostasis and correcting the HA phenotype in the absence of humoral immunity against the transgenic protein.

Anti-CD117 mAb conjugated to the ribosome-inactivating protein toxin saporin (sap) was utilized to create a potent HSC-targeted immunotoxin to condition bone marrow in preparation for HSCT gene therapy. Other studies have demonstrated the effectiveness of this antibody-toxin combination^34^, ^35^, ^36^ or CD45-sap immunotoxin^36^, ^37^, ^38^ for non-genotoxic conditioning in traditional HSCT models. We have found CD117-sap to be more effective than CD45-sap in enabling specific HSC depletion and donor cell engraftment in pilot studies with HA and C57BL/6 mice (Figure S12). The more restricted expression of CD117 compared with CD45 in the hematopoietic compartment enabled the use of a 6-fold lower dose (0.5 mg/kg versus 3.0 mg/kg) of CD117-sap to achieve robust HSC depletion in the absence of prolonged lymphopenia, making it more cost effective as well as limiting exposure of the recipient to the drug-toxin conjugate. Due to its lack of a lectin B-chain, and therefore significantly diminished ability to enter cells unilaterally, saporin has a high safety profile.^39^ However, since vascular leak syndrome has been previously identified as a dose-limiting toxicity in immunotoxin clinical trials,^40^ administration of a minimal dose is favorable.

Published reports describing the application of non-genotoxic antibody-based conditioning for HSPC-directed gene therapy are limited. In a recent study, a combination of CD117-, CD45-, and CD8-sap immunotoxins was necessary to achieve engraftment of fVIII-modified HSCs and sustained platelet-confined fVIII expression.^41^ To our knowledge, only one report has described the use of a single-agent CD117-targeting immunotoxin to facilitate engraftment of LV-modified HSPCs.^42^ Autologous CD34^+^ HSPCs from rhesus macaques were modified with a β-globin construct and successfully engrafted under CD117-amanitin conditioning. In contrast to our study, these studies did not involve production of a secreted, foreign therapeutic gene product that is known to be highly immunogenic (i.e., fVIII in HA), a unique challenge that requires the induction of sufficient immune tolerance to support lifelong expression of a new circulating protein. Nevertheless, observations from this preliminary nonhuman primate study are encouraging evidence that CD117-based immunotoxin conditioning may be safely and effectively translated to human gene therapy trials in the near future.

In the current study, host immune suppression was found to be a requirement for achieving engraftment of ET3 gene-modified HSPCs using CD117-sap conditioning. We initially sought to incorporate mATG into our non-genotoxic regimen. Human ATG products (e.g., Thymoglobulin, ATGAM) are US Food and Drug Administration (FDA)-approved hyperimmune therapeutics commonly used in the prevention of acute rejection of solid organ allografts^43^ and graft-versus-host disease (GVHD) in allogeneic HSCT.^44^ ATG is known to elicit rapid T cell depletion, although other potential mechanisms for immune suppression have been posited, and the full spectrum of its multifaceted mechanisms of action has yet to be completely elucidated.^45^ Nevertheless, ATG is an effective immune-suppressive agent in humans, and its use is incorporated into a current clinical trial of HSPC-directed LV gene therapy for HA (ClinicalTrials.gov identifier: NCT04418414). We found that the addition of mATG enabled long-term engraftment of ET3 gene-modified donor cells in the majority of HA mice conditioned with CD117-sap. Still, over 40% of recipients receiving CD117-sap + mATG did not exhibit sustained engraftment. ATG contains a wide variety of polyvalent antibodies with a broad range of binding specificities. Known target antigens include markers expressed on T and B lymphocytes but also on monocytes, dendritic cells, and endothelium.^30^ Some target antigens, such as CD45 and CD11b, are found on many different leukocyte populations, while HLA class I and II and β2 microglobulin expression is widespread even outside the hematopoietic compartment. Antibodies targeting CXCR4, an antigen present on HSCs, are also found in ATG.^29^ Although clinical-grade ATG products for human use are better characterized, the heterogeneous nature, incomplete characterization, and lot-to-lot variability of the mouse-specific ATG reagent may contribute to inconsistent outcomes in the setting of murine HSCT. Despite its promiscuous binding profile (Figures 4, S8, and S9), we found that mATG alone does not effectively deplete endogenous HSCs (Figure S3A) and therefore does not condition the bone marrow in a way that permits appreciable levels of gene-modified donor cell chimerism (Figures 2C and S5B). However, if residual mATG remains in circulation at the time of transplantation, it may bind receptors such as CXCR4 in the donor HSC graft. This could lead to either downregulation of surface expression (i.e., modulation, a known functional mechanism of ATG action^45^) or physical inhibition of chemotactic interactions by blocking the CXCR4/SDF-1 axis and disrupting donor HSC homing to bone marrow niches. We explored adjustments to the timing and frequency of mATG administration in HSCT studies and found that when mATG was administered on day −1 and on the day of transplantation, engraftment of donor HSPCs was significantly impaired (Figure S9C). Further, when ATG was administered 5 days before HSCT, with an additional dose given 3 days after HSCT, chimerism declined dramatically within the first 8 weeks. In corroboration of these findings, Jin et al.^46^ recently reported that rabbit anti-human ATG also binds human bone marrow cells and purified CD34^+^ human fetal liver cells *in vitro*. Moreover, human hematopoietic reconstitution in the periphery, as well as human cell engraftment in spleen and bone marrow, were completely abrogated when human ATG was administered to NSG mice 1 day after CD34^+^ cell transplantation. In contrast, graft failure did not occur in controls that received ATG 21 days after transplantation or PBS, providing further evidence that circulating ATG at the time of HSCT can interfere with HSC engraftment.^46^ Also in concordance with our observations (Figures 4B and S3A), Jin et al.^46^ found that although ATG can bind human HSPCs and impede engraftment, HSCs residing in bone marrow niches, though coated in ATG after administration, are resistant to depletion. In spite of these observations, to our knowledge there are no reports that show ATG interferes with HSPC engraftment in the setting of clinical human HSCT, and it is used widely and successfully in this context. Therefore, we believe the current findings underscore the critical importance of optimal timing of ATG administration. Indeed, in another recent study, Samelson-Jones et al.^47^ reported that appropriate timing of ATG administration was a critical factor in the prevention of humoral immunity against factor IX (fIX) in a nonhuman primate AAV2 gene therapy model. As a non-genotoxic conditioning regimen for HSCT gene therapy for HA is moved toward clinical translation, timing and dosage of human ATG will be thoroughly explored for its utility as part of this regimen in order to take advantage of its important immune suppressive actions while minimizing its ability to interfere with HSC engraftment.

Although human ATG remains a strong candidate as part of a clinically translatable non-genotoxic regimen, inconsistent outcomes in murine studies paired with a demonstrated, unequivocal requirement for immune suppression led us to refine our approach in the murine model to more precisely target the T cell-specific antigens CD4, CD8, and CD40L, as T cells are required for the development of anti-fVIII immunity. Chhabra et al.^48^ took a similar approach, using a regimen of four naked antibodies aimed at depleting host HSCs (CD117 [clone ACK2] and CD47 mAbs) and host T cells (CD4 and CD8 mAbs) to accomplish donor HSC engraftment in a murine model of minor major histocompatibility complex (MHC)-mismatched allogeneic HSCT. This approach was successful at accomplishing approximately 20% donor HSC engraftment but required at least 11 consecutive antibody injections and 3 donor HSPC transplants. A more recent study combined 1.5 mg/kg CD117-sap with CD4, CD8, and CD40L mAbs and rapamycin to achieve full MHC-mismatched HSCT using whole bone marrow.^35^ Though long-term donor LT-HSC chimerism reached only 3.4%, this was enough to establish donor-specific skin allograft tolerance. In our gene therapy model, we were able to accomplish an average of 75.1% bone marrow chimerism of donor LT-HSCs, with 100% of transplant recipients maintaining long-term engraftment, using a 3-fold lower dose of CD117-sap, enabling therapeutic transgene expression without immune rejection by administering only 3 doses of depleting agents (a single immunotoxin administration plus two injections of mAb cocktails) and one HSPC transplant. This protocol limits the immunotoxin dose and invasiveness of the procedure and minimizes potential risks associated with multiple injections—an important consideration for patients with HA.

For each component of this protocol, reagents with human activity will need to be identified to advance into clinical testing. There are many research-grade anti-human CD117 recombinant, humanized mAbs, single chain variable fragment (scFvs), or Fab fragments that are commercially available with known inhibitory activity of CD117 signaling (e.g., clones 19, Ab1, and Hum10) that can be tested for *in vivo* human CD34^+^ cell depletion. Several anti-human CD117 mAbs (e.g., LMJ729, CK6, and SR-1) have been tested in preclinical or clinical studies of HSCT conditioning or for cytotoxicity toward CD117-expressing cancers. A clinical trial testing an LMJ729-based antibody-drug conjugate (LOP628) for acute myelogenous leukemia (ClinicalTrials.gov identifier: NCT02221505) was discontinued in 2015 during phase 1 when three participants developed hypersensitivity reactions attributed to co-engagement of FcγR and CD117 present on mast cells.^49^ Promising preclinical results were later obtained by Shizuru and colleagues^50^ using SR-1 to deplete both normal and myelodysplastic syndrome (MDS) human HSCs in murine xenograft models, as well as endogenous HSPCs in cynomolgus macaques using the humanized version AMG191.^51^ A current phase 1 dose-escalation trial sponsored by Jasper Therapeutics testing JSP191 (formerly AMG191) as a conditioning agent in patients undergoing HSCT for severe combined immune deficiency (SCID) has shown encouraging early results for this indication, achieving up to 7% donor granulocyte chimerism 24 weeks post-transplantation (ClinicalTrials.gov identifier: NCT02963064).^52^ However, similar to the anti-mouse CD117 mAb clone ACK2, SR-1/JSP191 is a CD117 antagonist, accomplishing HSC depletion by blocking SCF signaling. Previous studies in mice have shown that while naked antagonistic CD117 mAbs are successful at achieving bone marrow conditioning and donor HSC engraftment in the setting of immune deficiency,^53^ appreciable donor engraftment in immunocompetent hosts using this approach required the addition of low-dose radiation^54^ or additional agents for CD47 blockade.^48^ Although varying levels of CD34^+^ HSPC depletion were demonstrated in immunocompetent nonhuman primates using JSP191, successful engraftment has not yet been reported. Endogenous HSPC depletion and subsequent HSPC engraftment using JSP191 could potentially be enhanced as a saporin-based immunotoxin for gene therapy applications in immunocompetent hosts. Another potential candidate for clinical development of an anti-human CD117 mAb is clone 104D2. Czechowicz et al.^34^ demonstrated the ability of a 104D2-saporin conjugate to eliminate the development of human myeloid cells in peripheral blood of mice xenografted with human umbilical cord blood HSCs up to 120 days post-conditioning, implying that this immunotoxin was successful at clearing human HSCs *in vivo*. Alternatively, clone 17F11 was shown to elicit robust downregulation of surface CD117 on MO7e cells in a similar fashion as its cognate ligand SCF,^55^ suggesting induction of receptor internalization, an important characteristic of antibodies that create potent immunotoxins. Clone 17F11 could also be explored for its efficacy as a human HSC-targeted immunotoxin.

Aside from human ATG, there are multiple avenues that could be explored for translation of the more targeted immune-suppressive component of our protocol. Alemtuzumab targets CD52 present on T and B cells, as well as monocytes, macrophages, natural killer (NK) cells, and some granulocytes. It is used clinically for the treatment of chronic lymphocytic leukemia and multiple sclerosis and has also been used as a T cell-depleting agent for GVHD prophylaxis or as an alternative to ATG in allogeneic HSCT settings.^56^, ^57^, ^58^, ^59^ Muromonab-CD3, an FDA-approved mouse mAb used to treat acute rejection of solid organ grafts, has also been used in bone marrow transplant settings.^60^, ^61^, ^62^ Similar to the mAb cocktail used in the current study, these drugs clear host T cells and may be effective in our HSCT gene therapy protocol. Alternatively, investigational mAbs that specifically target CD4 (e.g., MAX.16H5^63^^,^^64^ or IT1208^65^) or CD3^66^ (e.g., teplizumab, otelixizumab, visilizumab, or foralumab) might be considered. A similar approach to that taken in a recent phase 1/2 clinical trial of HSC-directed LV gene therapy for the primary immunodeficiency Wiskott-Aldrich syndrome, in which fludarabine and rituximab (CD20 mAb) were used to achieve immune cell depletion prior to transplantation, could be pursued.^11^^,^^67^

The addition of T cell costimulatory blockade to our regimen using CD40L mAbs ensured that if any residual T cells remained following CD4/CD8 mAb depletion and became activated through an encounter with ET3 protein, CD40L on these cells would be prevented from interacting with CD40 on B cells, dendritic cells, and other antigen-presenting cells, inhibiting humoral and cell-mediated immune responses to ET3. In a study of MHC-mismatched murine HSCT, Langford-Smith et al.^68^ found that blockade of both signal 1 (CD4/CD8 block) and signal 2 (CD40L block) was essential for long-term donor engraftment using non-myeloablative conditioning (low-dose busulfan); signal 1 or signal 2 blockade alone was insufficient to prevent rejection. In a xenograft model of T cell-mediated rejection of human CD34^+^ cells, Oh et al.^69^ demonstrated that costimulatory blockade using CTLA4-IgG1 (abatacept) in the early post-transplantation period prevented antigen-specific T cell alloreactivity and CD34^+^ rejection. CTLA4-Ig fusion proteins target a different costimulatory pathway, working as competitive inhibitors of CD28/B7 signaling, thereby promoting anergy in activated T cells. Two CTLA4-Ig drugs on the market, abatacept and belatacept, are clinically approved for treatment of rheumatoid arthritis and prophylaxis of renal transplant rejection, respectively. Abatacept in particular has been studied for its potential application as GVHD prophylaxis in various allogeneic HSCT settings,^70^, ^71^, ^72^ as well as for its ability to prevent anti-vector immune responses and allow repeated AAV8 administration in fIX gene therapy.^73^ Since thromboembolic complications in a phase 1 trial of human CD40L mAb therapy suspended clinical development of a CD40L-targeting agent for human use,^74^ newer mAbs are being developed and tested in clinical trials that disrupt CD40/CD40L signaling by instead targeting CD40 (e.g., bleselumab).^75^, ^76^, ^77^ As an alternative to costimulatory blocking agents, basiliximab, an interleukin (IL)-2 receptor antagonist that prevents T cell replication and T cell-dependent B cell activation, has been used in combination with ATG as GVHD prophylaxis^78^ and may be effective at suppressing anti-fVIII immune responses in our regimen.

In summary, we report successful implementation of a completely non-genotoxic antibody-based conditioning regimen to facilitate HSC-directed gene therapy in a preclinical murine model of HA. We developed a method for achieving high-level long-term engraftment of ET3-modified donor HSPCs, sustained curative levels of plasma procoagulant activity, phenotypic correction, and elimination of immunological rejection. The studies described herein demonstrated an explicit requirement for immune suppression to enable engraftment of ET3 gene-modified cells. Further studies will focus on the development of a human-specific non-genotoxic regimen that combines CD117 immunotoxin with non-genotoxic, clinically available immune-suppressive agents, such as ATG, alemtuzumab, and/or abatacept. Nevertheless, these findings establish strong proof of concept toward translation of a non-genotoxic approach to HSCT conditioning for HA gene therapy to the clinic in the future.

## Materials and methods

### Mice

Exon 16-disrupted HA mice^79^ backcrossed to C57BL/6 background were used as transplantation recipients in this study. Congenic donor mice were CD45.1^+^ (B6.SJL-Ptprc^a^ Pepc^b^/BoyJ; JAX stock #002014). Mouse strains were bred and maintained at an Emory University Division of Animal Resources facility. Male and female mice 7–10 weeks of age were used in this study. Transplant recipients within each experiment were age-matched and randomized to experimental groups. Animal studies were approved by Emory University Institutional Animal Care and Use Committee.

### Immunotoxin preparation and conditioning

Biotinylated anti-CD117 (clone 2B8, Biolegend, San Diego, CA, USA) was combined at a 1:1 molar ratio with either streptavidin-ZAP (ATS, San Diego, CA, USA) or an in-house-developed streptavidin-saporin product.^27^ CD117-saporin immunotoxin was delivered by retro-orbital injection at a dose of 0.5 mg/kg on day −5 before HSCT. Rabbit anti-mouse ATG (Cedarlane, Burlington, NC, USA) was administered by intraperitoneal injection at a dose of 30 mg/kg on day −5. Anti-CD4 (clone GK1.5, Bio X Cell, Lebanon, NH, USA) and anti-CD8 (clone YTS 169.4, Bio X Cell, Lebanon, NH, USA) were administered in 100 μg doses by intraperitoneal injection on day −2 and day 0. Anti-CD40L (clone MR-1, Bio X Cell, Lebanon, NH, USA) was administered in 500 μg doses by intraperitoneal injection on day 0. Irradiation controls were exposed to either 11 Gy cesium-137 or 9 Gy X-ray radiation in split doses on day 0. Further reagent details are provided in the Supplemental materials.

### Bone marrow depletion and flow cytometric analysis

Mice were conditioned with CD117-sap immunotoxin and sacrificed after 5 days. Femurs were dissected and crushed using a mortar and pestle. Bone marrow was strained through a 40 μm cell strainer and red blood cells (RBCs) lysed using Hybri-Max RBC lysis buffer (Sigma-Aldrich, St. Louis, MO, USA). Bone marrow leukocytes were stained for flow cytometric detection of HSPCs using the following LSK-SLAM markers: lineage cocktail, CD117 (c-Kit), Sca-1, CD48, and CD150. Data were acquired using a BD LSR II (San Jose, CA, USA) or Cytek Aurora (Fremont, CA, USA) flow cytometer. Flow cytometric analysis was performed using FlowJo v10.7.1 (BD, Ashland, OR, USA). Further reagent details are provided in the Supplemental materials.

### Histology

Fresh tissues were isolated and fixed in 10% neutral buffered formalin for 24–48 h and then transferred to 70% ethanol before paraffin embedding. For acute toxicity studies, HA mice were conditioned with CD117-sap immunotoxin and sacrificed after 5 days. Paraffin-embedded sections were stained with H&E and tissue morphology evaluated for signs of toxicity or pathological lesions by a veterinary pathologist. For long-term morphological analysis, tissues were collected from HA mice that had received immunotoxin conditioning and gene therapy with sustained ET3 expression after 34 weeks. H&E-stained paraffin-embedded sections were evaluated for normal tissue morphology after long-term engraftment by a veterinary pathologist. Histology services were provided by the Histology and Molecular Pathology Laboratory at Yerkes National Primate Research Center (Atlanta, GA, USA).

### Sca-1^+^ cell isolation, *ex vivo* expansion, transduction, and transplantation

Sca-1^+^ isolation from murine bone marrow was performed by immunomagnetic bead selection as previously described.^80^ Briefly, bone marrow from CD45.1^+^ donor mice was harvested and incubated with a biotinylated anti-Sca-1 antibody (Biolegend, San Diego, CA, USA) followed by incubation with anti-biotin microbeads (Miltenyi, Gaithersburg, MD, USA). Labeled cells were then passed through an LS column loaded on a MidiMACS Separator (Miltenyi). Sca-1^+^ cells were harvested and cultured in serum-free StemSpan medium (Stem Cell Technologies, Vancouver, BC, Canada) for 3 days in the presence of mouse SCF (100 ng/mL), mouse IL-3 (20 ng/mL), human IL-11 (100 ng/mL), and human Flt-3 ligand (100 ng/mL). All cytokines were purchased from R&D Systems (Minneapolis, MN, USA). Sca-1^+^ cells were transduced on day −1 and day 0 with half volume CD68-ECO-ET3-LV (LV production described in detail by Lytle et al.^8^) at a density of 2.0 × 10^6^ Sca-1^+^ cells/mL and multiplicity of infection (MOI) of 13–63. Transduced CD45.1^+^ Sca-1^+^ HSPCs were harvested, washed, and resuspended in PBS and then transplanted by retro-orbital injection into preconditioned HA mice.

### Peripheral blood collection and analysis

Peripheral blood was periodically collected from the retro-orbital sinus in 3.8% w/v sodium citrate anticoagulant. CBC analyses were performed using a HemaTrue Veterinary Hematology Analyzer (Heska, Loveland, CO, USA). Remaining whole blood was centrifuged at 2,000 × *g* for 15 min at 4°C. Plasma was removed and frozen at −80°C until used for downstream analyses. Cell pellets were lysed using Hybri-Max RBC lysis buffer (Sigma-Aldrich, St. Louis, MO, USA), and peripheral blood leukocytes were stained for flow cytometric detection of donor- and host-derived cells using monoclonal antibodies against the following surface markers: CD45.1, CD45.2, CD3, B220 (CD45R), and Gr-1 (Ly-6G/Ly-6C). Data were acquired using a BD LSR II (San Jose, CA, USA) or Cytek Aurora (Fremont, CA, USA) flow cytometer. Flow cytometric analysis was performed using FlowJo v10.7.1 (BD, Ashland, OR, USA). Further reagent details are provided in the Supplemental materials.

### ET3 activity measurement

ET3 activity was measured in plasma samples using the Chromogenix Coatest SP Factor VIII chromogenic assay kit (Diapharma, West Chester Township, OH, USA) according to manufacturer’s instructions. Standard curves were generated using Factor Assay Control Plasma (George King Bio-Medical, Overland Park, KS, USA). Absorbance at 405 nm was measured kinetically using a VersaMax Microplate Reader (Molecular Devices, San Jose, CA, USA).

### Vector copy number measurement

Vector copy number in DNA samples isolated from peripheral blood, spleen, and bone marrow was determined by quantitative real-time polymerase chain reaction using primers specific for Rev response element (RRE) sequences present in the CD68-ECO-ET3-LV construct as previously described.^18^ Briefly, genomic DNA (gDNA) was isolated, and 50 or 100 ng DNA was analyzed in triplicate by generating a 1× Power SYBR Green Master Mix (Thermo-Fisher, Waltham, MA, USA) containing 250 nM primers. Standard curve was generated by analyzing serial dilutions of RRE plasmid DNA suspended in 50 ng HEK293T/27 gDNA as background. Quantitative real-time polymerase chain reaction measurements were performed on an Applied Biosystems 7500 Real-Time PCR System (Foster City, CA, USA).

### Anti-ET3 ELISA assay

Mouse plasma was analyzed for the presence of anti-ET3 IgG by ELISA. Microtiter plates were coated with 1.5 μg/mL ET3 antigen in coating buffer (20 mM bicine, 2 mM CaCl_2_ [pH 9.0]) overnight at 4°C. Plates were washed twice with wash buffer (20 mM HEPES, 0.15 M NaCl, 2 mM CaCl_2_, 0.05% Tween 20, 0.05% sodium azide [pH 7.4]) and then incubated in blocking buffer (wash buffer plus 2.0% bovine serum albumin) overnight at 4°C. Plates were washed twice with wash buffer immediately before the assay. Mouse plasma samples were diluted 1:20 in blocking buffer, and 25 μL plasma dilution was added to ET3-coated wells. Plates were incubated at room temperature for 1 h and then washed twice with wash buffer. Goat anti-mouse IgG-alkaline phosphatase (AP) was diluted 1:1,000 and 25 μL added to each well. Plates were incubated at room temperature for 1 h and then washed twice with wash buffer. AP substrate was reconstituted according to manufacturer’s instructions (AP Substrate Kit, cat. #1721063, Bio-Rad, Hercules, CA, USA), and 40 μL was added to each well. Plates were incubated at room temperature for 20 min, and reaction was then quenched by adding 0.4 M NaOH. Absorbance at 405 nm was measured using a VersaMax Microplate Reader (Molecular Devices, San Jose, CA, USA).

### Modified Bethesda assay

Plasma was tested for inhibitory activity against ET3 using a modification of the Bethesda assay^81^ as previously described.^18^ Briefly, fVIII-deficient plasma was reconstituted with ET3 to a final concentration of 0.8–1.2 IU/mL and kept on ice until use. Equal volumes of reconstituted plasma and test plasma were incubated for 2 h at 37°C, followed by measurement of residual ET3 activity using the one-stage coagulation assay. Residual ET3 activity was calculated by dividing the ET3 activity in the test sample by that of the reconstituted plasma sample. Dilutions were identified that produced at least two values in the range between 40% and 60% residual activities. One Bethesda unit (BU) per milliliter is defined as the dilution of inhibitor that produces 50% inhibition of fVIII activity using the published reference curve for interpolation.^81^

### Phenotypic correction tail snip assay

Phenotypic correction of the HA bleeding phenotype was assessed using a quantitative tail snip assay as previously described.^82^ Briefly, 15 mL conical tubes containing 13 mL PBS were weighed before the procedure and placed in a 37°C water bath. Mice were anesthetized with isoflurane and a transection made 4 mm from the distal tip of the tail. Snipped tails were immediately placed in pre-weighed PBS tubes at 37°C, and bleeding into the tube was allowed for 40 min. Tubes were capped and weighed at the conclusion of the assay, and the amount of blood lost from each mouse was calculated. Mice were euthanized upon completion of the procedure.

### ATG binding studies

HA mice were injected intraperitoneally with either 30 mg/kg rabbit anti-mouse ATG (Cedarlane, Burlington, NC, USA) or PBS. After 4 h, mice were sacrificed and peripheral blood, bone marrow, and spleen cells were isolated. Single-cell suspensions from each tissue were incubated with mouse Fc block, followed by mouse anti-rabbit IgG secondary antibody to detect the presence of surface-bound ATG antibodies. Cells were then washed and stained with an LSK-SLAM flow cytometry panel for bone marrow cells or a mature lineage marker panel for blood and spleen cells. Data were acquired using a Cytek Aurora (Fremont, CA, USA) flow cytometer. Flow cytometric analysis was performed using FlowJo v10.7.1 (BD, Ashland, OR, USA). Further reagent details are provided in the Supplemental materials.

### Statistical analysis

Shapiro-Wilk test was used for normality testing of continuous variables. When comparing two groups, Student’s t test or Mann-Whitney U test was used depending on normality of data. When comparing more than two groups, one-way ANOVA with Tukey’s multiple comparison tests was performed for normally distributed data, and Kruskal-Wallis test with Dunn’s multiple comparison test was performed for nonparametric analyses. Data are reported as mean ± sample SD. Statistical analysis was performed using GraphPad Prism v9.0.0 (San Diego, CA, USA).

## Supplemental information

Document S1. Figures S1–S12 and Supplemental materials

Document S2. Article plus supplemental information
