# Supplementary material for: Non-genotoxic conditioning facilitates hematopoietic stem cell gene therapy for hemophilia A using bioengineered factor VIII
Source: Mol Ther Methods Clin Dev. 2021 May 5;21:710–27. doi: 10.1016/j.omtm.2021.04.016 (PMC8181577; doi:10.1016/j.omtm.2021.04.016)
Supplement: Document S1. Figures S1–S12 and Supplemental materials [file mmc1.pdf]

OMTM, Volume *21*

Supplemental information

Non-genotoxic conditioning facilitates hematopoietic stem cell gene therapy for hemophilia A using bioengineered factor VIII

Athena L. Russell, Chengyu Prince, Taran S. Lundgren, Kristopher A. Knight, Gabriela Denning, Jordan S. Alexander, Jaquelyn T. Zoine, H. Trent Spencer, Shanmuganathan Chandrakasan, and Christopher B. Doering

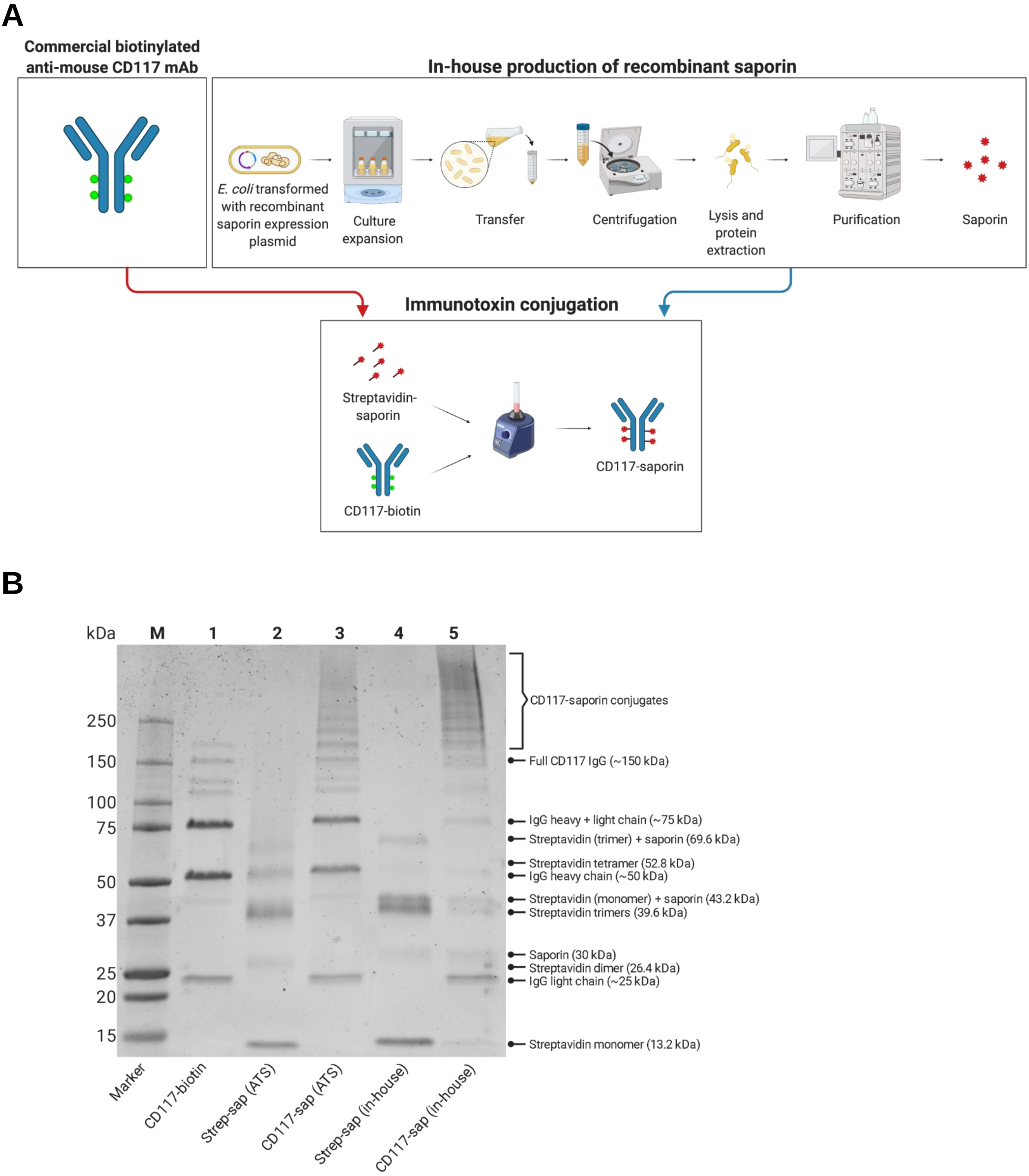

Supplemental Figure 1: (A) Recombinant saporin was generated and bioconjugated to streptavidin in-house. Streptavidin-saporin (strep-sap) was then linked to biotinylated CD117 mAb to produce the final CD117-saporin immunotoxin product. **(B)** Coomassie-stained SDS-PAGE shows CD117 (2B8) mAb, commercial strep-sap, in-house recombinant strep-sap, and their associated CD117-sap conjugates.

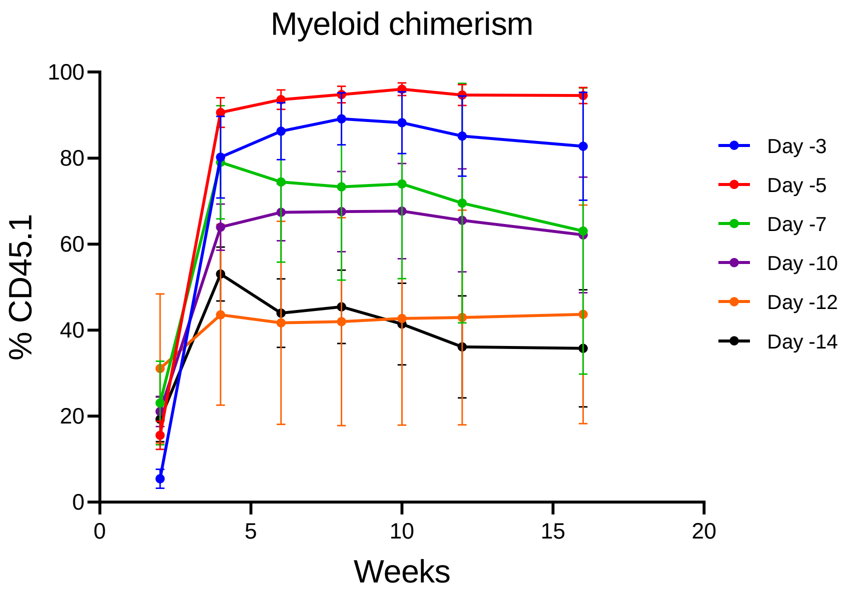

Supplemental Figure 2: C57BL/6 mice were conditioned with 0.5 mg/kg CD117-sap at various time points, and then transplanted with 5 x 10^6 CD45.1$^+$ whole bone marrow cells. Optimal engraftment kinetics in the myeloid compartment were attained when immunotoxin was administered 5 days prior to transplantation. Data represent mean ± sample SD.

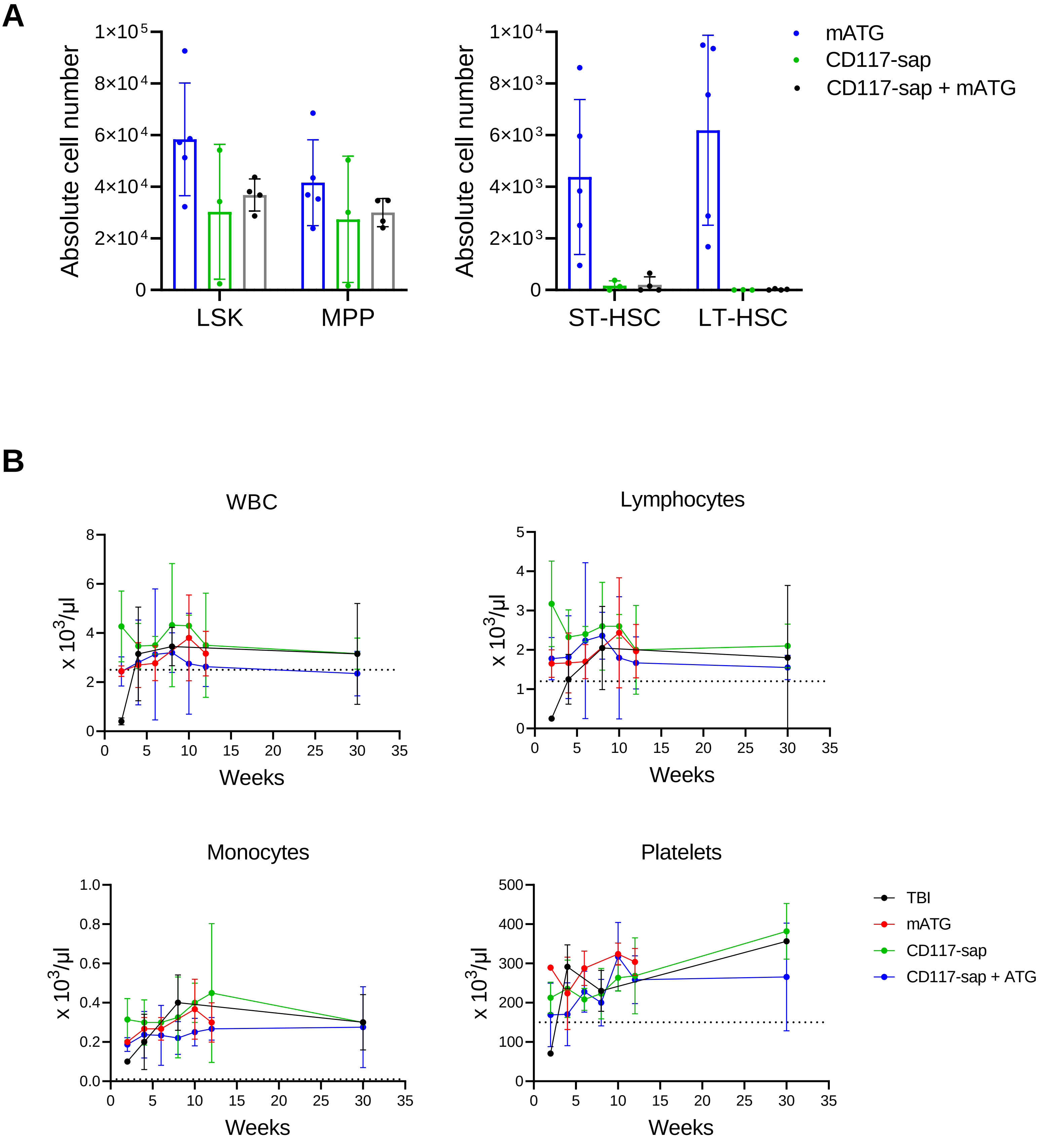

Supplemental Figure 3: (A) ST-HSC and LT-HSC compartments are selectively and robustly depleted after CD117-sap or CD117-sap + mATG conditioning. mATG alone did not produce depletion of HSPC or HSC compartments. **(B)** Treatment with mATG (n = 3), CD117-sap (n = 7) or CD117-sap + mATG (n = 7) did not produce prolonged cytopenias. Mice treated with TBI (n = 2) were included to ensure viability and engraftment potential of HSPCs following isolation, ex vivo culture and transduction. TBI mice exhibited leukopenia and thrombocytopenia until 4 weeks post-transplantation. See also Figure 2B.

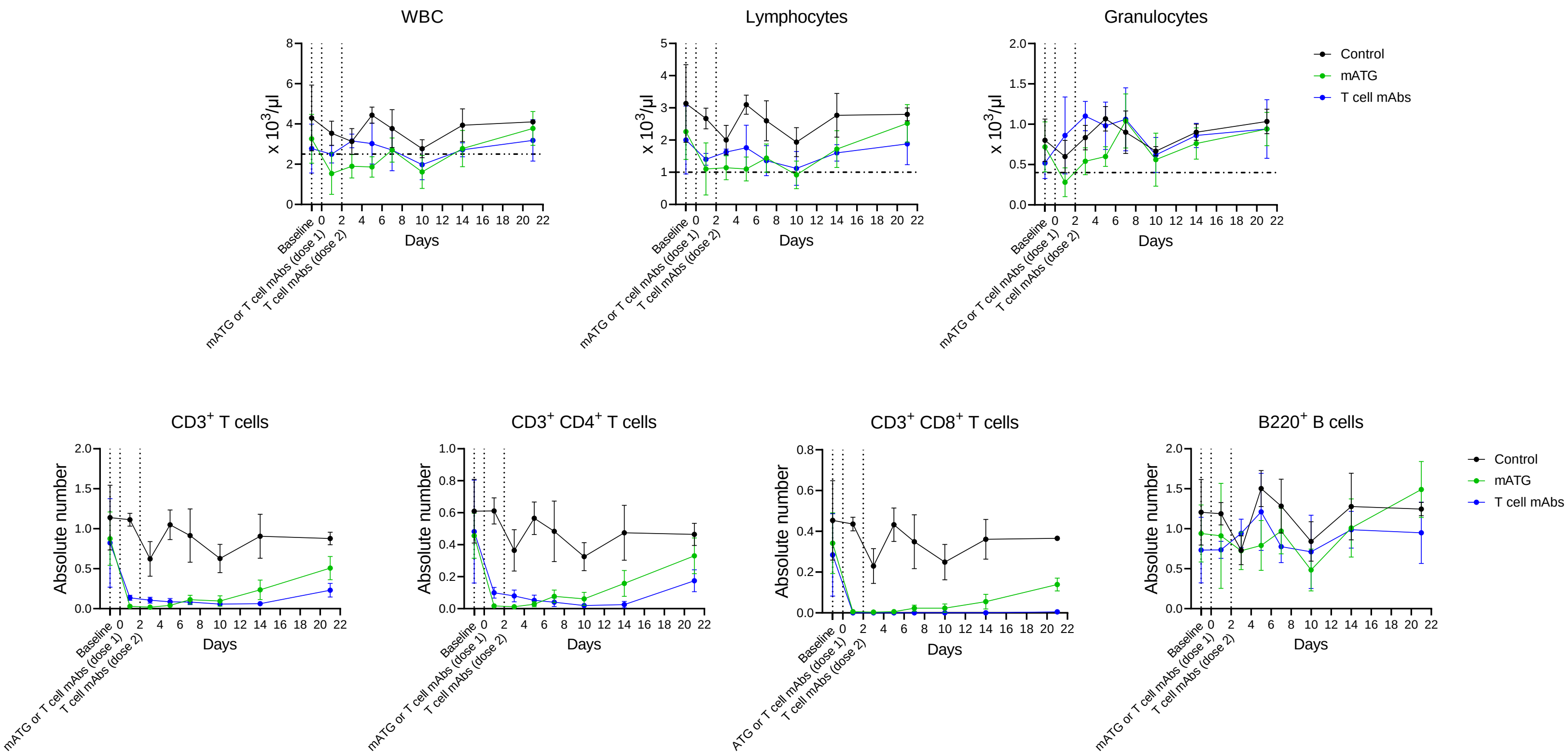

Supplemental Figure 4: HA mice were injected intraperitoneally with either 30 mg/kg mATG or the T cell mAbs regimen (dosing and schedule as described in Materials and Methods). Peripheral blood was collected periodically for 21 days to follow the kinetics of depletion and recovery in WBC and lymphocyte compartments resulting from antibody immune suppression. Horizontal dotted line represents lower limit of reference range for respective populations. See also Figure 2B and 5B.

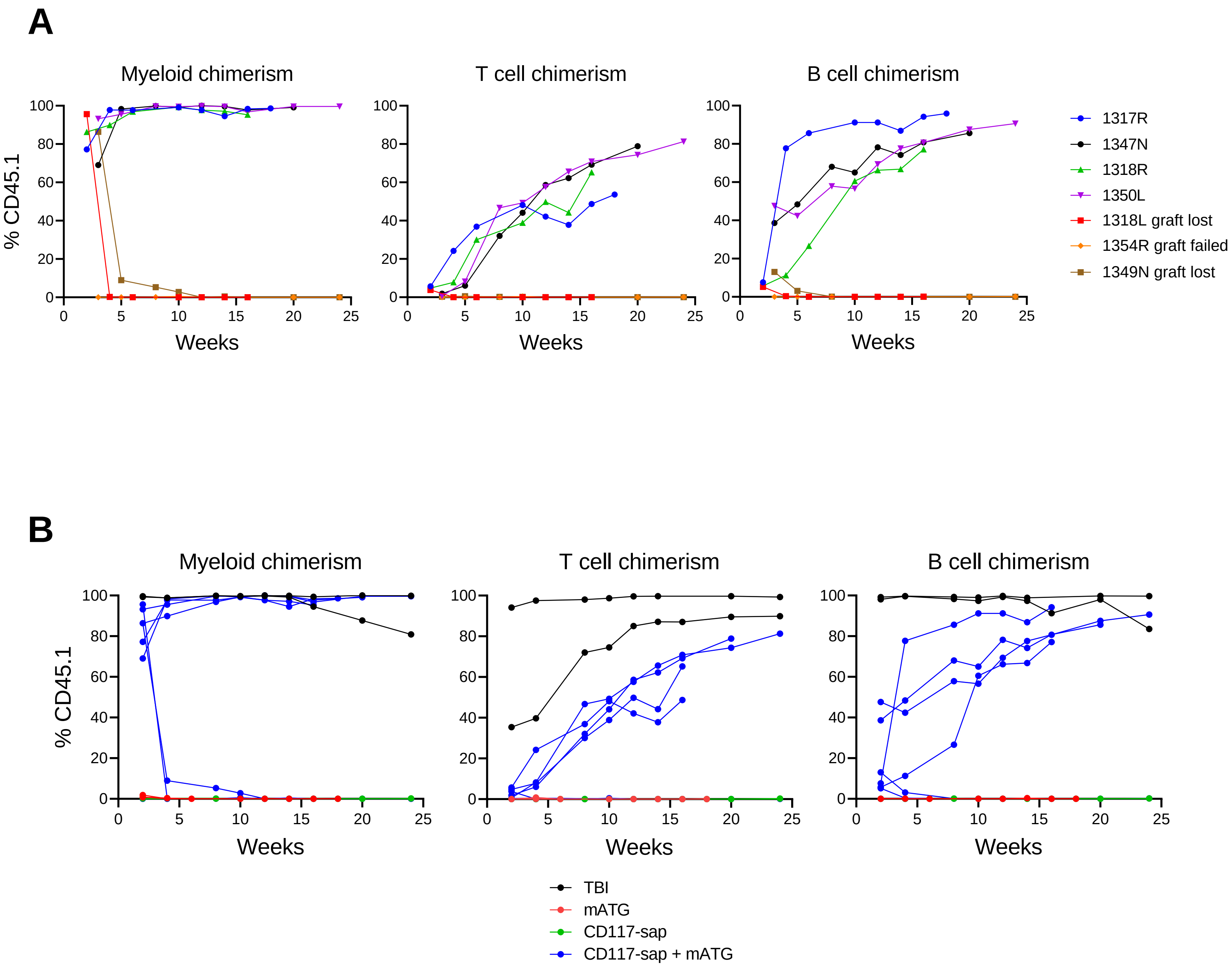

Supplemental Figure 5: (A) Two weeks after transplantation, 1 of 7 mice (across two independent experimental cohorts) conditioned with CD117-sap + mATG failed to engraft ET3 gene-modified HSPCs (orange diamond), while the remaining six mice achieved early myeloid chimerism of 72.5 ± 33.3% (mean ± sample SD). By four weeks post-transplantation, myeloid chimerism levels decreased from 95.6% to 0.22% and 86.4% to 8.94% in two additional mice conditioned with CD117-sap + mATG (red square and brown square). **(B)** Mice treated with TBI (n = 2) were included to ensure viability and engraftment potential of HSPCs following isolation, ex vivo culture and transduction. See also Figure 2C.

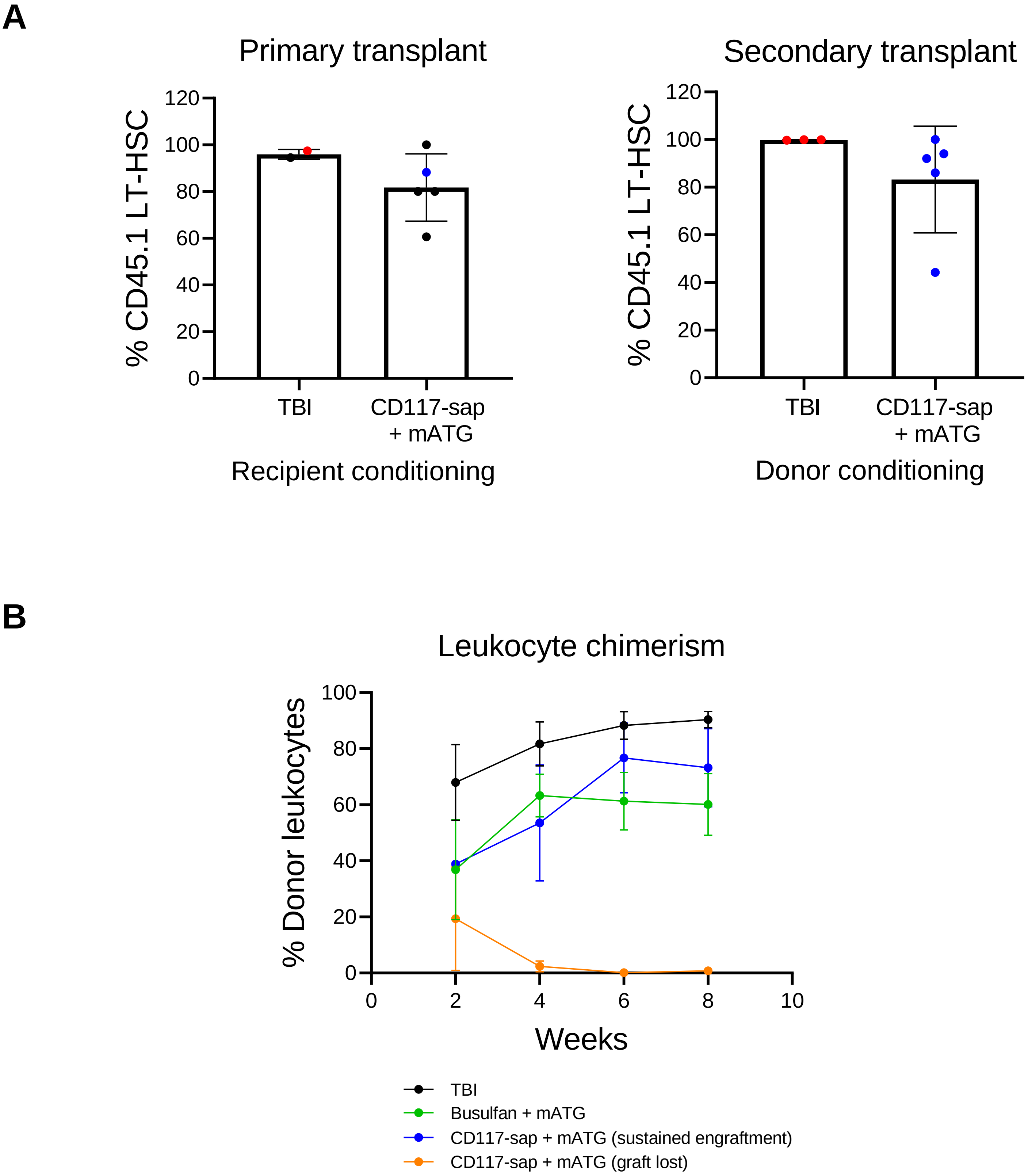

Supplemental Figure 6: (A) Engraftment of donor-derived LT-HSCs in chimeric HA mice conditioned with CD117-sap + mATG was 80.2 ± 16.1% (primary transplant). Mice treated with TBI (n = 2) were included to ensure viability and engraftment potential of HSPCs following isolation, ex vivo culture and transduction. Donor-derived LT-HSCs in TBI mice was 95.9 ± 2.1%. Bone marrow from one primary recipient conditioned with CD117-sap + mATG (blue dot, left panel) and one conditioned with TBI (red dot, left panel) were harvested and transplanted into lethally irradiated secondary HA recipients (n = 5 secondary recipients per donor). $CD45.1^{+}$ LT-HSC engraftment was 87.2 ± 19.6% in secondary recipients receiving CD117-sap + mATG-conditioned bone marrow (blue dots, right panel) and 99.9 ± 0.06% for secondary recipients receiving TBI-conditioned bone marrow (red dots, right panel). See also Figure 2D. **(B)** High donor leukocyte chimerism after CD68-ECO-ET3-LV HSCT is achievable using TBI (n = 9) or non-myeloablative conditioning (busulfan + mATG or CD117-sap + mATG) (n = 4-5). Three mice conditioned with CD117-sap + mATG did not maintain long-term engraftment of ET3 gene-modified cells (orange). Data represent mean ± sample SD.

A

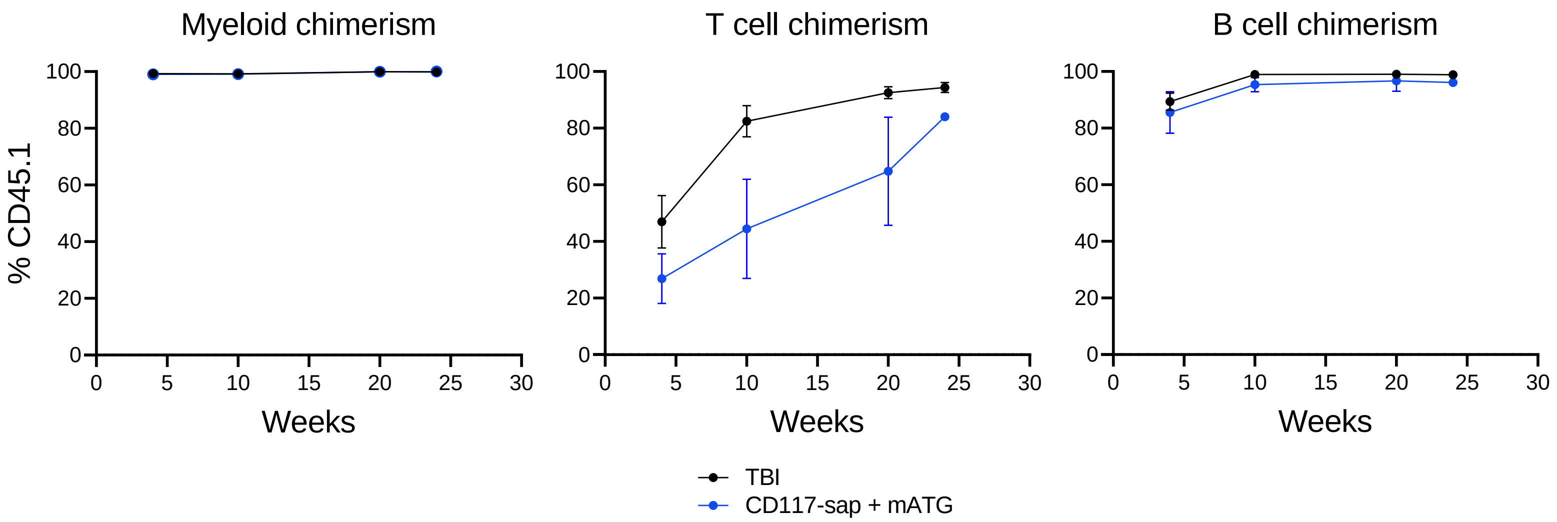

B

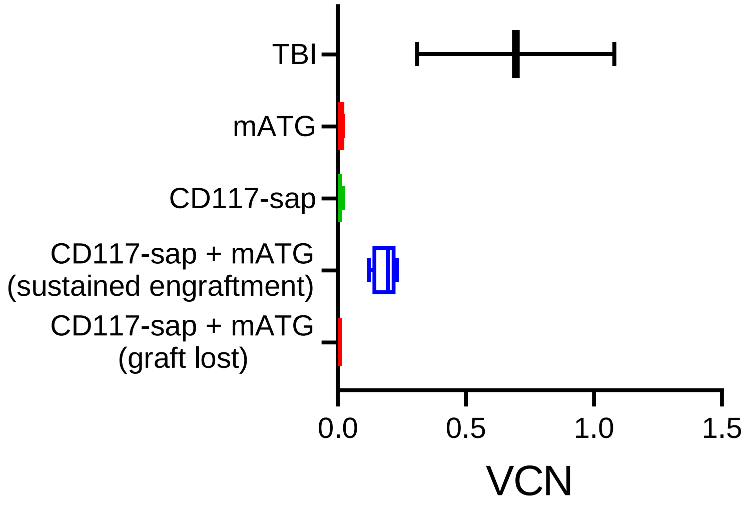

C

Bethesda Titers in Mice That Developed ET3 Inhibitors

Conditioning	Outcome	Titer (BU/ml)
CD117-sap (mouse 1)	No engraftment	50
CD117-sap (mouse 2)	No engraftment	44
CD117-sap + mATG	86.4% myeloid engraftment, followed by rejection beginning 4 weeks post-transplantation	37

Supplemental Figure 7: (A) High-level multilineage chimerism in secondary recipients after TBI or CD117-sap + mATG conditioning indicates engraftment of donor LT-HSCs in primary recipients. (n = 5 in each group) Data represent mean ± sample SD. See also Figure 2E. **(B)** Mice treated with TBI (n = 2) were included to ensure viability and engraftment potential of HSPCs following isolation, ex vivo culture and transduction. Peripheral blood VCN in mice conditioned with CD117-sap + mATG was 0.19 ± 0.05 copies per genome and 0.70 ± 0.54 copies per genome in mice conditioned with TBI. Proviral DNA was undetectable in mice treated with mATG alone, CD117-sap alone, or mice that lost engraftment under CD117-sap + mATG conditioning. See also Figure 3B. **(C)** Modified Bethesda assay was used to measure inhibitor titers of mice from Figure 3C at 24 weeks post-transplantation.

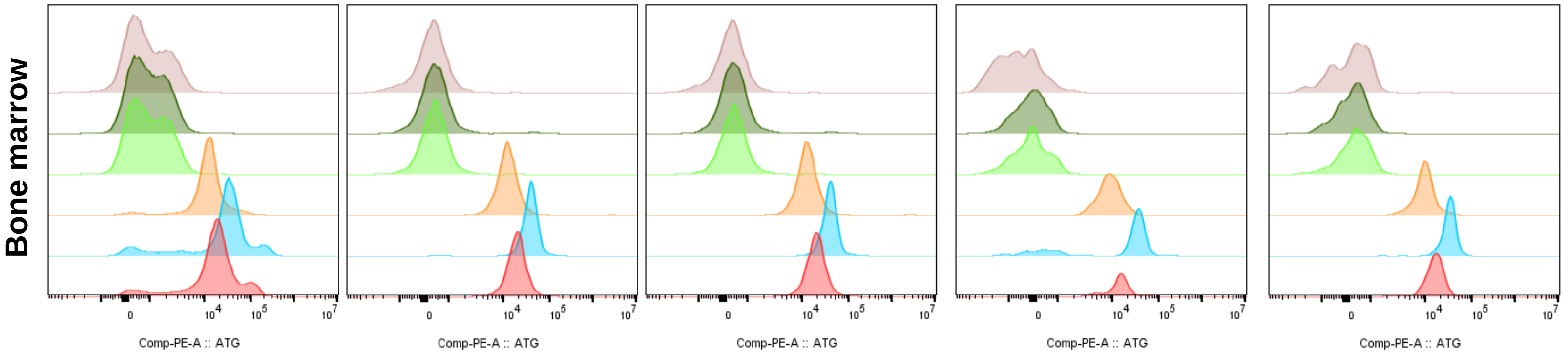

	Sample Name	Subset Name	Median : Comp-PE-A
	PBS1.fcs	Lin-	699
	PBS2.fcs	Lin-	850
	PBS3.fcs	Lin-	853
	ATG1.fcs	Lin-	11834
	ATG2.fcs	Lin-	30109
	ATG3.fcs	Lin-	16858

	Sample Name	Subset Name	Median : Comp-PE-A
	PBS1.fcs	LSK	293
	PBS2.fcs	LSK	402
	PBS3.fcs	LSK	391
	ATG1.fcs	LSK	11795
	ATG2.fcs	LSK	36941
	ATG3.fcs	LSK	18574

	Sample Name	Subset Name	Median : Comp-PE-A
	PBS1.fcs	Q1: CD150- , CD48+	295
	PBS2.fcs	Q1: CD150- , CD48+	411
	PBS3.fcs	Q1: CD150- , CD48+	391
	ATG1.fcs	Q1: CD150- , CD48+	12238
	ATG2.fcs	Q1: CD150- , CD48+	38160
	ATG3.fcs	Q1: CD150- , CD48+	18966

	Sample Name	Subset Name	Median : Comp-PE-A
	PBS1.fcs	Q4: CD150- , CD48-	-610
	PBS2.fcs	Q4: CD150- , CD48-	-32.0
	PBS3.fcs	Q4: CD150- , CD48-	-90.7
	ATG1.fcs	Q4: CD150- , CD48-	8138
	ATG2.fcs	Q4: CD150- , CD48-	30652
	ATG3.fcs	Q4: CD150- , CD48-	14397

	Sample Name	Subset Name	Median : Comp-PE-A
	PBS1.fcs	Q3: CD150+ , CD48-	312
	PBS2.fcs	Q3: CD150+ , CD48-	263
	PBS3.fcs	Q3: CD150+ , CD48-	339
	ATG1.fcs	Q3: CD150+ , CD48-	9264
	ATG2.fcs	Q3: CD150+ , CD48-	31429
	ATG3.fcs	Q3: CD150+ , CD48-	15416

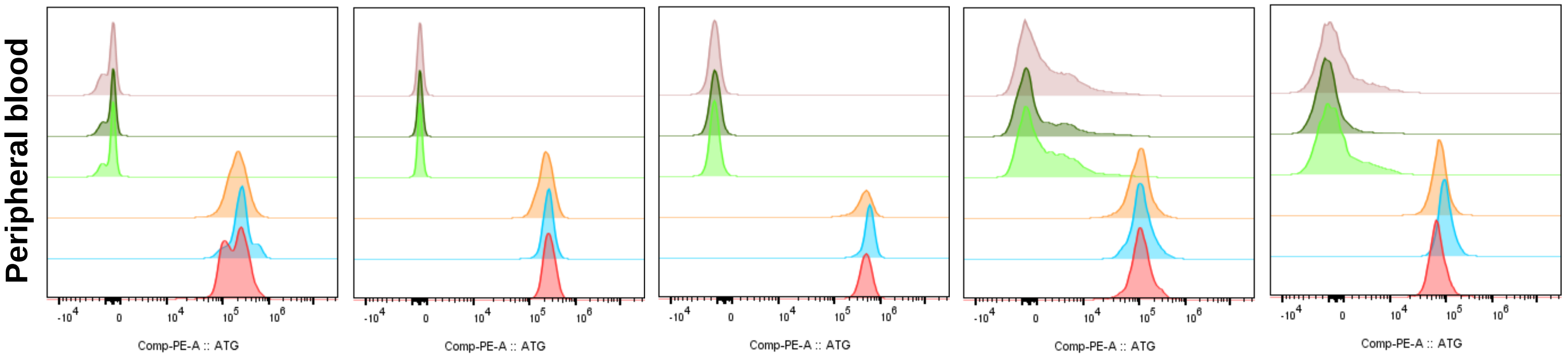

	Sample Name	Subset Name	Median : Comp-PE-A
	PBS1.fcs	Q1: B220- , CD3+	-272
	PBS2.fcs	Q1: B220- , CD3+	-169
	PBS3.fcs	Q1: B220- , CD3+	-169
	ATG1.fcs	Q1: B220- , CD3+	195586
	ATG2.fcs	Q1: B220- , CD3+	236420
	ATG3.fcs	Q1: B220- , CD3+	182384

	Sample Name	Subset Name	Median : Comp-PE-A
	PBS1.fcs	CD3+ CD4+	-37.6
	PBS2.fcs	CD3+ CD4+	-37.6
	PBS3.fcs	CD3+ CD4+	-47.0
	ATG1.fcs	CD3+ CD4+	211261
	ATG2.fcs	CD3+ CD4+	240622
	ATG3.fcs	CD3+ CD4+	244902

	Sample Name	Subset Name	Median : Comp-PE-A
	PBS1.fcs	CD3+ CD8+	-1449
	PBS2.fcs	CD3+ CD8+	-1401
	PBS3.fcs	CD3+ CD8+	-1439
	ATG1.fcs	CD3+ CD8+	4.31E5
	ATG2.fcs	CD3+ CD8+	5.40E5
	ATG3.fcs	CD3+ CD8+	4.55E5

	Sample Name	Subset Name	Median : Comp-PE-A
	PBS1.fcs	Q3: B220+ , CD3-	885
	PBS2.fcs	Q3: B220+ , CD3-	-18.8
	PBS3.fcs	Q3: B220+ , CD3-	829
	ATG1.fcs	Q3: B220+ , CD3-	96601
	ATG2.fcs	Q3: B220+ , CD3-	104767
	ATG3.fcs	Q3: B220+ , CD3-	102658

	Sample Name	Subset Name	Median : Comp-PE-A
	PBS1.fcs	Myeloid	-536
	PBS2.fcs	Myeloid	-1295
	PBS3.fcs	Myeloid	-517
	ATG1.fcs	Myeloid	69930
	ATG2.fcs	Myeloid	90924
	ATG3.fcs	Myeloid	62369

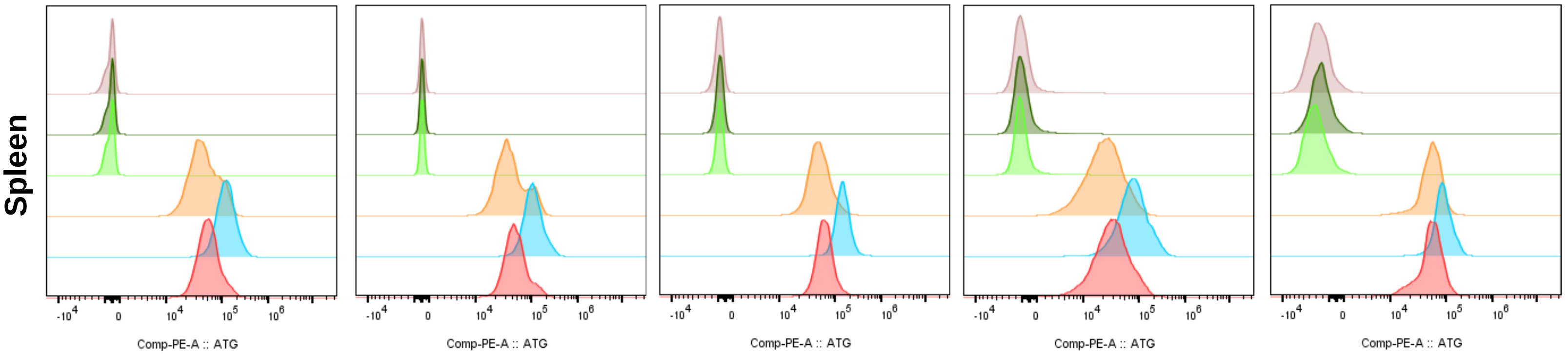

	Sample Name	Subset Name	Median : Comp-PE-A
	PBS1.fcs	Q1: B220- , CD3+	-225
	PBS2.fcs	Q1: B220- , CD3+	-207
	PBS3.fcs	Q1: B220- , CD3+	-254
	ATG1.fcs	Q1: B220- , CD3+	40251
	ATG2.fcs	Q1: B220- , CD3+	116022
	ATG3.fcs	Q1: B220- , CD3+	50283

	Sample Name	Subset Name	Median : Comp-PE-A
	PBS1.fcs	CD3+ CD4+	-28.2
	PBS2.fcs	CD3+ CD4+	-28.2
	PBS3.fcs	CD3+ CD4+	-28.2
	ATG1.fcs	CD3+ CD4+	34651
	ATG2.fcs	CD3+ CD4+	101621
	ATG3.fcs	CD3+ CD4+	44463

	Sample Name	Subset Name	Median : Comp-PE-A
	PBS1.fcs	CD3+ CD8+	-914
	PBS2.fcs	CD3+ CD8+	-838
	PBS3.fcs	CD3+ CD8+	-838
	ATG1.fcs	CD3+ CD8+	45449
	ATG2.fcs	CD3+ CD8+	136773
	ATG3.fcs	CD3+ CD8+	58079

	Sample Name	Subset Name	Median : Comp-PE-A
	PBS1.fcs	Q3: B220+ , CD3-	-1171
	PBS2.fcs	Q3: B220+ , CD3-	-1142
	PBS3.fcs	Q3: B220+ , CD3-	-1247
	ATG1.fcs	Q3: B220+ , CD3-	21158
	ATG2.fcs	Q3: B220+ , CD3-	71795
	ATG3.fcs	Q3: B220+ , CD3-	28220

	Sample Name	Subset Name	Median : Comp-PE-A
	PBS1.fcs	Myeloid	-2526
	PBS2.fcs	Myeloid	-2193
	PBS3.fcs	Myeloid	-3057
	ATG1.fcs	Myeloid	49806
	ATG2.fcs	Myeloid	81969
	ATG3.fcs	Myeloid	49178

Supplemental Figure 8: Flow cytometric analysis revealed measurable mATG binding in vivo to all primitive and mature hematopoietic cell populations examined. (n = 3 in each group) See also Figure 4.

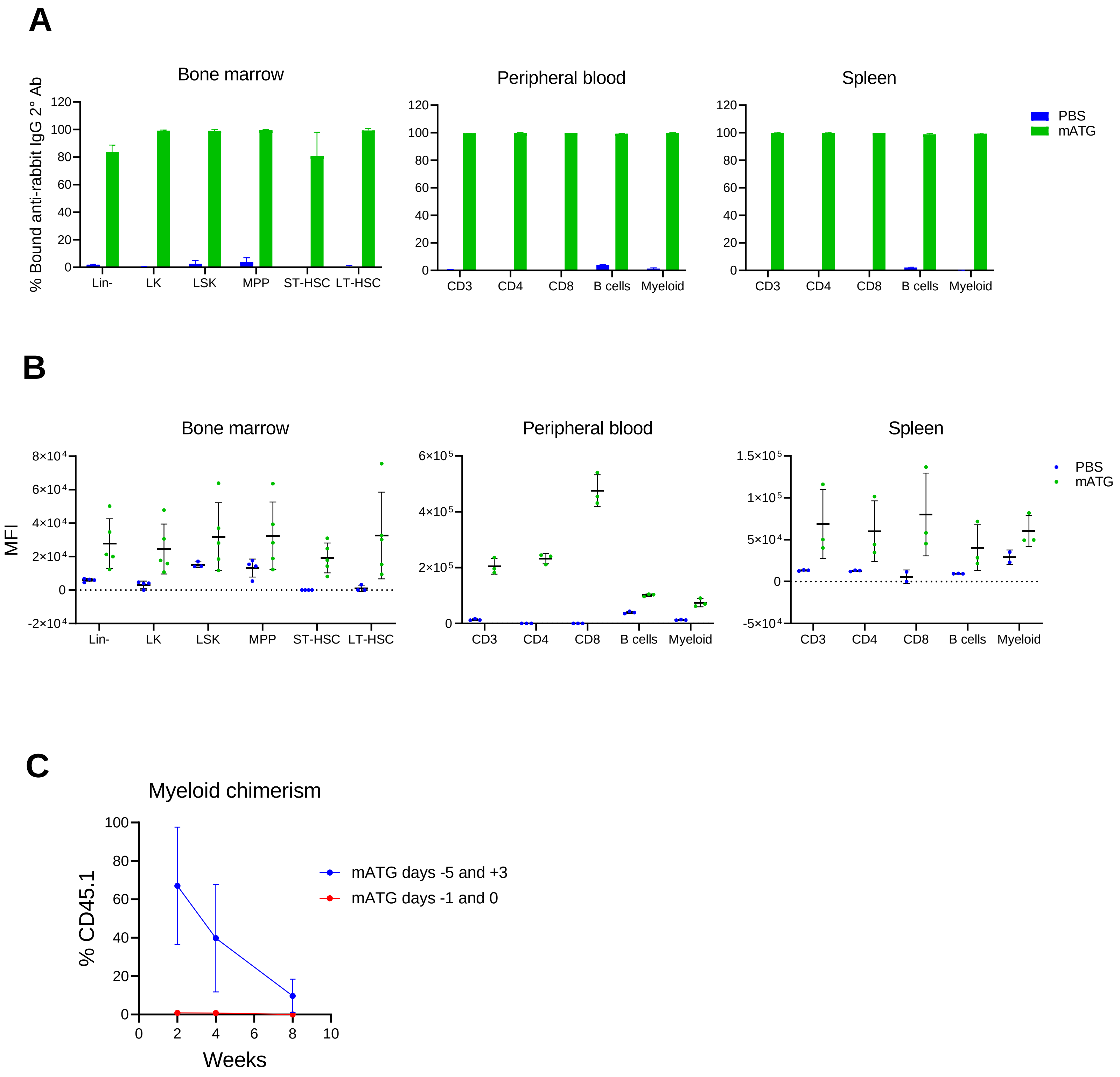

Supplemental Figure 9: **(A and B)** In vitro binding studies using mATG and bone marrow, peripheral blood and spleen cells mirrored results observed in vivo (see Figure 4A and 4B). **(C)** HSCT-LV studies were performed to test alternative timing and dosing schedules of mATG. Two doses of mATG given at days -5 and +3 resulted in early chimerism that was eventually lost. Two doses of mATG given at days -1 and 0 resulted in complete abrogation of engraftment.

A

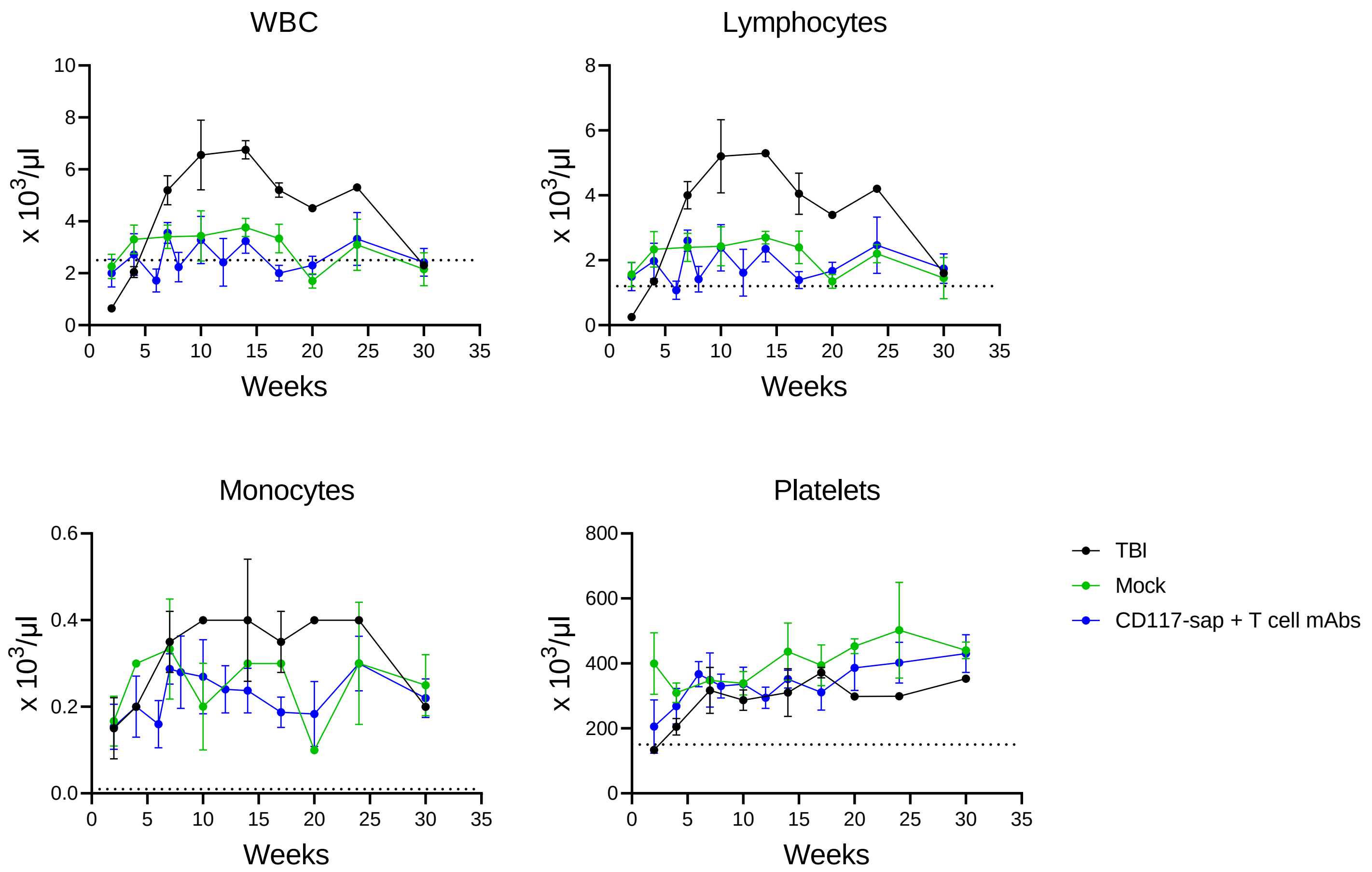

B

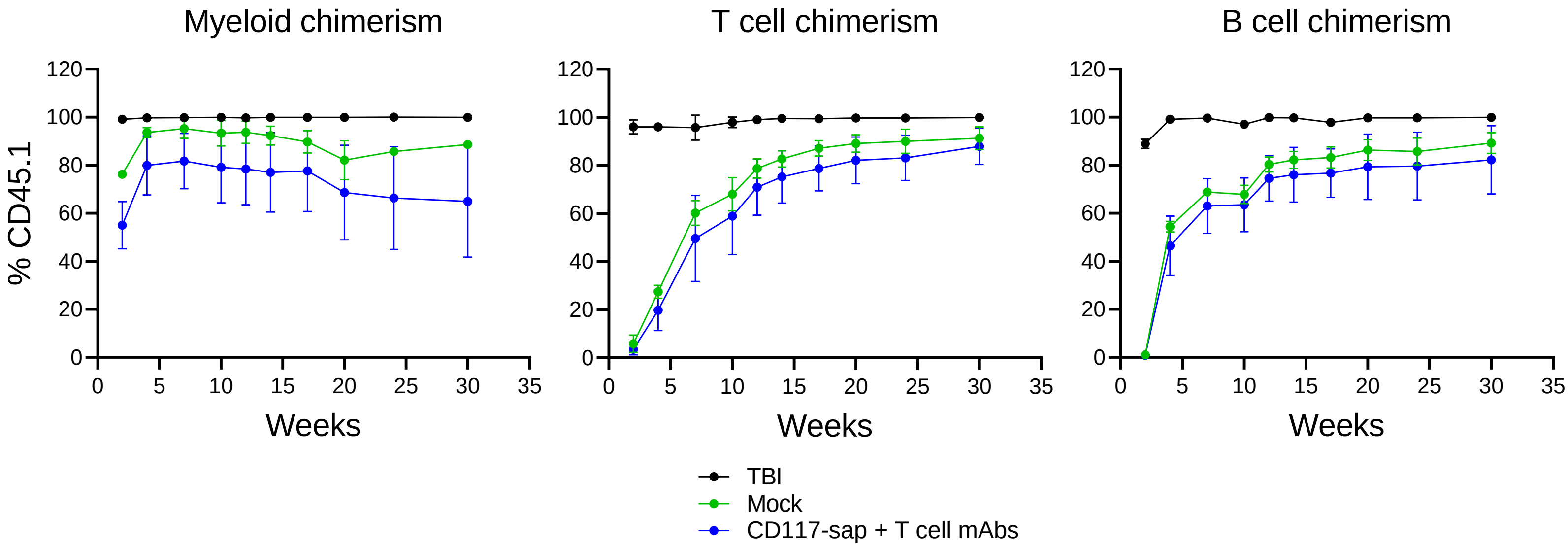

Supplemental Figure 10. (A) Lymphocytes, monocytes and platelets measured in the normal range by 2 weeks post-transplantation in CD117-sap + T cell mAbs conditioned mice that received either ET3-modified cells or mock transduced HSPCs. Mice treated with TBI (n = 2) were included to ensure viability and engraftment potential of HSPCs following isolation, ex vivo culture and transduction. TBI mice had low lymphocytes and platelets until 4 weeks post-transplantation. See also Figure 5B. **(B)** High-level multilineage chimerism was achieved in primary recipients following TBI (n = 2), CD117-sap + T cell mAbs (n = 13), or CD117-sap + T cell mAbs and mock transduced HSCT (n = 3). Data represent mean ± sample SD. See also Figure 5C.

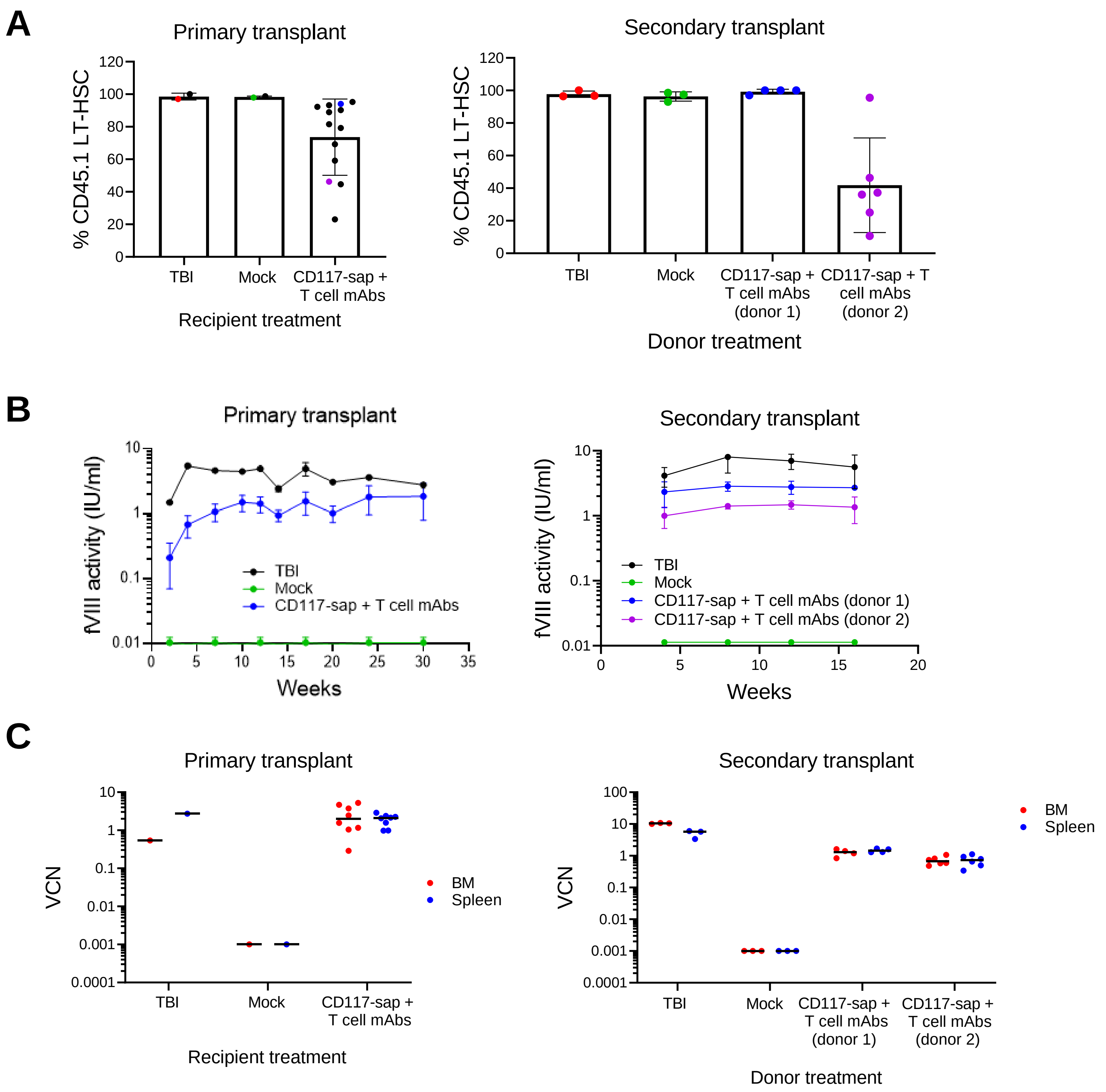

Supplemental Figure 11: (A) Engraftment of donor-derived CD68-ECO-ET3-LV transduced LT-HSCs in chimeric HA mice conditioned with CD117-sap + T cell mAbs was 73.6 ± 23.5% (range: 23.0 – 95.2%). Bone marrow from two primary recipients that received CD117-sap + T cell mAbs conditioning and ET3-modified HSPCs (LT-HSC chimerism 94.1% and 46.2%; blue and purple dots, left panel) was harvested and transplanted into lethally irradiated secondary HA recipients (n = 6). CD45.1^{+} LT-HSC engraftment in secondary recipients measured 99.3 ± 1.5% in recipients of donor 1 (blue dots, right panel) and 41.8 ± 29.1% in recipients of donor 2 (purple dots, right panel). Mice treated with TBI (n = 2) were included to ensure viability and engraftment potential of HSPCs following isolation, ex vivo culture and transduction. LT-HSC engraftment in secondary recipients of TBI-treated mice (n = 3) and mice that received mock transduced cells (n = 3) was consistent with primary recipients (red and green dots, respectively). See also Figure 5D. **(B)** Curative ET3 levels of 0.49 ± 0.31 IU/ml were achieved in CD117-sap + T cell mAbs conditioned mice, steadily increasing to 1.76 ± 1.0 IU/ml by 30 weeks. No ET3 activity was detected in animals receiving mock transduced cells. ET3 activity in secondary recipients of donor 1 bone marrow reached 2.70 ± 0.24 IU/ml and 1.35 ± 0.59 IU/ml in secondary recipients of donor 2 marrow. Mice treated with TBI for the primary transplant were included to ensure viability and engraftment potential of HSPCs following isolation, ex vivo culture and transduction (n = 2 primary recipients, n = 3 secondary recipients). See also Figures 6A and 6C. **(C)** Copies of CD68-ECO-ET3-LV proviral DNA were measured from bone marrow and spleen. In mice receiving the CD117-sap + T cell mAbs regimen, VCN measured 2.5 ± 1.8 copies per genome in bone marrow and 1.9 ± 0.68 in spleen. For secondary recipients of CD117-sap + T cell mAbs treated mice, copies of CD68-ECO-ET3-LV proviral DNA in bone marrow measured 1.3 ± 0.33 (donor 1) and 0.71 ± 0.21 (donor 2) and spleen measured 1.47 ± 0.20 (donor 1) and 0.72 ± 0.29 (donor 2). No proviral DNA was measured in animals transplanted with mock transduced cells. See also Figure 6B.

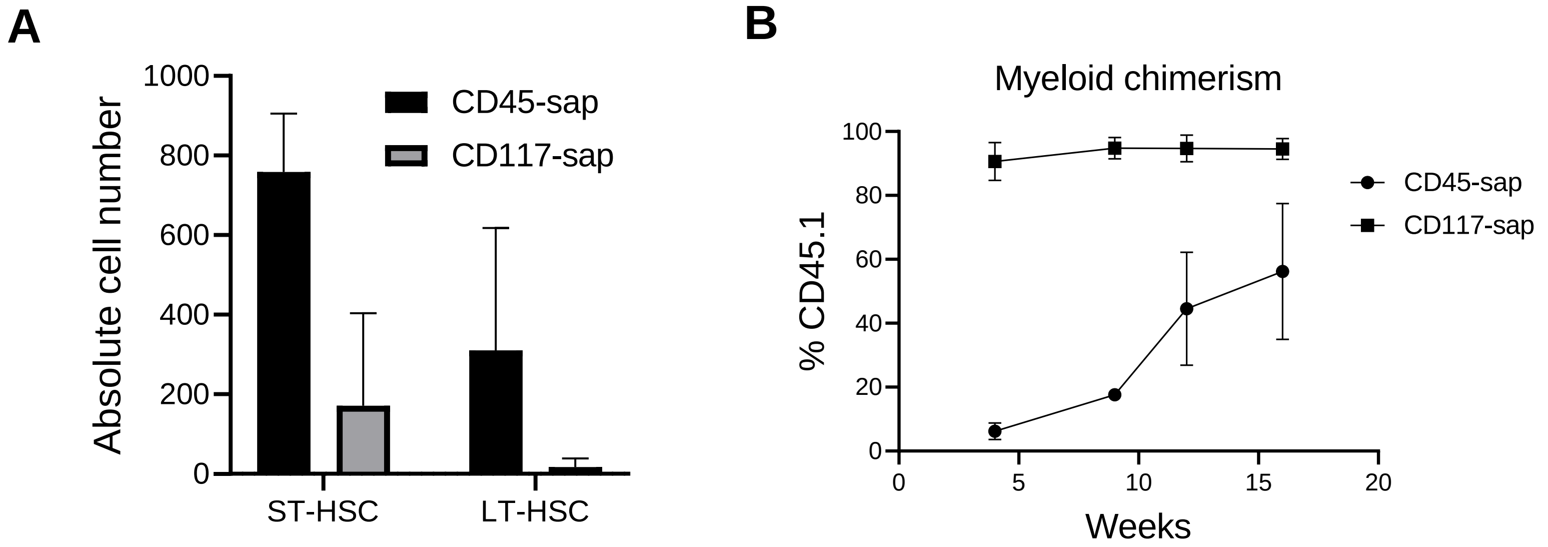

Supplemental Figure 12: (A) In pilot studies, HA mice were conditioned with 0.5 mg/kg CD117-sap or 3.0 mg/kg CD45-sap and sacrificed after 7 days. Bone marrow was harvested, and depletion of ST-HSC and LT-HSC populations was analyzed by flow cytometry. **(B)** A pilot study was performed in which C57BL/6 mice were conditioned with either 0.5 mg/kg CD117-sap or 3.0 mg/kg CD45-sap, and then transplanted with 1 x 10^7 $CD45.1^+$ whole bone marrow cells after 5 days. Donor myeloid chimerism was consistently higher when CD117-sap was used.

Supplemental Materials

Conditioning reagents

Antigen/reagent	Conjugate	Clone	Manufacturer	Catalog number
CD117 (c-kit)	Biotin	2B8	Biolegend	105804
ZAP (saporin)	Streptavidin	NA	ATS	IT-27
CD4	NA	GK1.5	Bio X Cell	BE0003-1
CD8a	NA	YTS169.4	Bio X Cell	BE0117
CD40L	NA	MR-1	Bio X Cell	BE0017-1
Rabbit anti-mouse anti-thymocyte globulin	NA	NA	Cedarlane	CLAG3940T

Bone marrow flow cytometry antibodies

Antigen/reagent	Conjugate	Clone	Manufacturer	Catalog number
Lineage cocktail	FITC	NA	Biolegend	78022
CD117 (c-kit)	APC	ACK2	Biolegend	135108
Sca-1 (Ly-6A/E)	PE-Cy7	D7	Biolegend	108114
CD48	APC-Cy7	HM48-1	Biolegend	103432
CD150	BV421	Q38-480	BD	562811
CD45.1	PE	A20	BD	553776
CD45.2	Alexa Fluor 594	104	Biolegend	109850

Peripheral blood flow cytometry antibodies

Antigen/reagent	Conjugate	Clone	Manufacturer	Catalog number
CD45.1	PE	A20	BD	553776
CD45.2	APC	104	BD	558702
CD3	V450	500A2	BD	560801
CD45R/B220	PerCP	RA3-6B2	Biolegend	103234
Gr-1 (Ly-6G/Ly-6C)	BV605	RB6-8C5	Biolegend	108440

Spleen flow cytometry antibodies

Antigen/reagent	Conjugate	Clone	Manufacturer	Catalog number
CD3	V450	500A2	BD	560801
CD4	FITC	RM4-5	BD	553046
CD8a	PE	53-6.7	BD	553032
CD45R/B220	PE-Cy7	RA3-6B2	BD	552772
Gr-1 (Ly-6G/Ly-6C)	BV605	RB6-8C5	Biolegend	108440

ATG binding studies antibodies

Antigen/reagent	Conjugate	Clone	Manufacturer	Catalog number
Rabbit anti-mouse anti-thymocyte globulin	NA	NA	Cedarlane	CLAG3940T
Mouse anti-rabbit IgG	PE	NA	Santa Cruz Biotechnology	sc-3753
